# Cardiac Computed Tomography in Infective Endocarditis: A Radiological Perspective on Structural Complications and Emerging Diagnostic Technologies

**DOI:** 10.3390/tomography12100145

**Published:** 2026-10-01

**Authors:** Loredana Elisabeta Popa, Bianca Boca, Dragoș Tudor Jula, Raluca Tomoaia, Călin Schiau, Paul-Andrei Ștefan, Raluca Rancea, Ruxandra Ștefana Beyer, Adrian Molnar, Monica Lupșor-Platon

**Affiliations:** 1 Department of Radiology and Medical Imaging, Nuclear Medicine, Faculty of Medicine, “Iuliu Hațieganu” University of Medicine and Pharmacy, 400012 Cluj-Napoca, Romania; paplory@yahoo.com (L.E.P.); monica.lupsor@umfcluj.ro (M.L.-P.); 2 Faculty of Biology, University of Bucharest, 91–95 Splaiul Independenței, Sector 5, 050095 Bucharest, Romania; 3 4th Department of Internal Medicine, Faculty of Medicine, “Iuliu Hațieganu” University of Medicine and Pharmacy, 400347 Cluj-Napoca, Romania; tomoaia.raluca@elearn.umfcluj.ro; 4 Department of Cardiology, Rehabilitation Hospital, 400012 Cluj-Napoca, Romania; 5 Department of Radiology and Medical Imaging, Faculty of Medicine, “Iuliu Hațieganu” University of Medicine and Pharmacy, 400012 Cluj-Napoca, Romania; calin.schiau@yahoo.com; 6 Department of Radiology, Emergency Clinical County Hospital Cluj-Napoca, 400006 Cluj-Napoca, Romania; 7 Department No. 1, Fundamental Disciplines, Faculty of Nursing and Healthcare Sciences, “Iuliu Hațieganu” University of Medicine and Pharmacy, 400012 Cluj-Napoca, Romania; paulandreistefan@gmail.com; 8 Radiology and Imaging Department, County Emergency Hospital, Clinicilor Street 3–5, 400006 Cluj-Napoca, Romania; 9 Department of Cardiology, “Niculae Stăncioiu” Heart Institute, 400001 Cluj-Napoca, Romania; raluca_rancea@yahoo.com (R.R.); anda_bogdan@yahoo.com (R.Ș.B.); 10 “Iuliu Hațieganu” University of Medicine and Pharmacy, 400012 Cluj-Napoca, Romania; 11 Department of Cardiovascular and Thoracic Surgery, “Iuliu Hațieganu” University of Medicine and Pharmacy, 400012 Cluj-Napoca, Romania; adrianmolnar097@gmail.com; 12 Department of Cardiovascular Surgery, “Niculae Stăncioiu” Heart Institute, 400001 Cluj-Napoca, Romania; 13 Department of Medical Imaging, Regional Institute of Gastroenterology and Hepatology “Prof. Dr. Octavian Fodor”, 400162 Cluj-Napoca, Romania

**Keywords:** infective endocarditis, cardiac computed tomography, prosthetic valve endocarditis, multimodality imaging, structural complications, transesophageal echocardiography, photon-counting detector CT, artificial intelligence, radiomics

## Abstract

**Background:** Infective endocarditis (IE) remains a life-threatening disease associated with substantial morbidity and mortality. Transthoracic and transesophageal echocardiography are the first-line imaging modalities, but their diagnostic performance may be reduced in patients with prosthetic valves, intracardiac devices, previous cardiac surgery, extensive calcification, or suspected periannular extension. Cardiac computed tomography (CT) provides complementary high-resolution anatomical information and has gained increasing importance within contemporary multimodality diagnostic pathways. **Scope:** This Review critically examines the current role of cardiac CT in IE, including acquisition and reconstruction strategies, lesion-specific imaging findings, applications in native, prosthetic, transcatheter, and device-related infection, extracardiac assessment, and preoperative planning. Comparative observations regarding echocardiography and selected multimodality techniques are integrated where directly relevant. Emerging applications of spectral CT, photon-counting detector CT, deep learning reconstruction, radiomics, and artificial intelligence are appraised according to four levels of evidence: clinically validated IE applications, preliminary IE-specific evidence, indirect cardiovascular evidence, and conceptual applications. **Main Findings:** Cardiac CT is particularly valuable for detecting and anatomically characterizing periannular complications, including abscesses, pseudoaneurysms, fistulous tracts, inflammatory extension, and prosthetic dehiscence. Multiphase electrocardiography (ECG)-gated acquisition improves the assessment of dynamic valvular and prosthetic abnormalities. Transesophageal echocardiography remains more sensitive for small or highly mobile vegetations, leaflet perforation, regurgitant jets, and intracardiac flow abnormalities, demonstrating that the two techniques are complementary rather than interchangeable. Cardiac CT also contributes to coronary assessment, surgical planning, evaluation of previous grafts and reconstructive material, and selected extracardiac complications. **Conclusions:** Cardiac CT is an important component of contemporary multimodality IE assessment, but it should not be regarded as a replacement for echocardiography. Its clinical value depends on the infected substrate, suspected complication, acquisition protocol, and integration with echocardiographic, microbiological, surgical, and clinical findings. Emerging technologies may improve image quality and quantitative characterization, but most remain supported by preliminary IE-specific, indirect cardiovascular, or conceptual evidence and should not yet be considered established IE-specific diagnostic tools.

## Simple Summary

Infective endocarditis is a serious infection that can damage heart valves and the surrounding tissues. Ultrasound of the heart remains the main imaging method used to diagnose the disease, but it may not always show the full extent of infection, particularly in patients with artificial heart valves, implanted cardiac devices, or previous heart surgery. This review explains how cardiac computed tomography can complement ultrasound by providing detailed three-dimensional information about valve damage, abscesses, abnormal cavities or connections, and complications around artificial valves. It also describes how computed tomography can assist with the assessment of complications outside the heart and with planning cardiac surgery. Newer imaging and artificial intelligence technologies are also discussed, although evidence supporting their routine use in infective endocarditis remains limited. Cardiac computed tomography should therefore be used as part of a combined imaging approach rather than as a replacement for ultrasound.

## 1. Introduction

Infective endocarditis (IE) is an infection of the endocardial surface of the heart that most commonly involves native or prosthetic valves but may also affect intracardiac devices and adjacent cardiovascular structures. Although relatively uncommon, IE has become increasingly frequent in several contemporary populations, a trend associated with population ageing, the growing prevalence of prosthetic heart valves and cardiac implantable electronic devices, the expansion of transcatheter valve interventions, and greater exposure to invasive healthcare procedures [1,2,3,4,5]. In 2019, the estimated global incidence of IE was 13.8 cases per 100,000 persons per year [5]. The epidemiological profile of IE has consequently shifted from disease predominantly associated with rheumatic valve pathology and streptococcal infection toward one increasingly affecting older and medically complex patients, with *Staphylococcus aureus* occupying a prominent etiological role [2,3,4]. Despite advances in microbiological diagnosis, antimicrobial therapy, cardiac surgery, and imaging, IE remains associated with substantial morbidity and mortality, and surgical intervention is required in a considerable proportion of patients [3,4,5].

The clinical presentation of IE is highly heterogeneous, ranging from fulminant sepsis and acute valvular destruction to subacute disease with nonspecific constitutional symptoms. Heart failure, embolic events, persistent infection, conduction abnormalities, and metastatic infectious complications may all be part of the initial presentation [3,4,5]. Diagnosis therefore requires integration of clinical findings, microbiological evidence, and imaging signs of endocardial or periannular involvement. The original Duke criteria, introduced in 1994 and modified in 2000, established a standardized framework for classifying IE as definite, possible, or rejected. However, their diagnostic performance is reduced in patients with prosthetic or transcatheter valves, intracardiac devices, previous cardiac surgery, and anatomically complex infection, in whom conventional imaging may remain negative or equivocal despite persistent clinical suspicion [4,5,6,7].

The growing contribution of advanced imaging has led to a substantial evolution of contemporary diagnostic frameworks. The 2015 European Society of Cardiology (ESC) criteria incorporated paravalvular lesions identified by cardiac computed tomography (CT) and abnormal periprosthetic activity detected by ^18^F-fluorodeoxyglucose positron emission tomography/computed tomography (^18^F-FDG PET/CT) or radiolabeled leukocyte single-photon emission computed tomography/computed tomography (WBC-SPECT/CT) as major imaging evidence in defined clinical settings [8]. The 2023 ESC and Duke–International Society for Cardiovascular Infectious Diseases (Duke-ISCVID) criteria further expanded the recognized anatomical, metabolic, molecular, microbiological, and surgical evidence [5,6]. In an external validation cohort of 595 patients with suspected IE, the 2023 Duke-ISCVID criteria improved sensitivity relative to the modified Duke criteria while maintaining high specificity [7]. A separate validation study similarly reported a moderate improvement in sensitivity without a significant reduction in specificity compared with previous diagnostic frameworks [9]. These developments reflect a broader transition from an echocardiography-dominated approach toward patient-specific multimodality assessment.

Transthoracic echocardiography (TTE) and transesophageal echocardiography (TEE) remain the first-line imaging modalities in suspected IE. They provide real-time assessments of valvular morphology, vegetation mobility, leaflet destruction, regurgitation, prosthetic instability, and hemodynamic consequences. TEE benefits from close proximity to the cardiac valves and posterior structures and is particularly effective for detecting small or highly mobile vegetations, leaflet perforation, paravalvular regurgitation, and intracardiac flow abnormalities. Nevertheless, echocardiographic evaluation may be limited by poor acoustic windows, extensive calcification, prosthetic material, previous reconstructive surgery, and device-related shadowing. TEE is also semi-invasive and may be contraindicated, poorly tolerated, or insufficient for determining the complete three-dimensional extent of periannular infection [10,11,12].

ECG-synchronized cardiac CT provides high-resolution three-dimensional assessment of valvular, periannular, periprosthetic, coronary, and postoperative anatomy. Its principal incremental value lies in defining the anatomical extent of infection and supporting procedural planning when echocardiography is inconclusive or anatomically limited [10,11,12,13,14]. Detailed lesion-specific comparisons between cardiac CT and TEE are presented in Section 5.

Beyond lesion detection, cardiac CT can support surgical planning by defining the relationship of infection to the coronary ostia, aortic root, intervalvular fibrosa, bypass grafts, prosthetic material, and structures relevant to repeat sternotomy [11,12,13,14]. Appropriate extension of the acquisition may also identify pulmonary or systemic embolic complications and other extracardiac manifestations. In prosthetic-valve, transcatheter-valve, and device-related infection, CT can be integrated with ^18^F-FDG PET/CT or radiolabeled leukocyte SPECT/CT to combine high-resolution anatomical information with evidence of active inflammatory or infectious activity. Nuclear imaging is particularly valuable in prosthetic and device-related disease and for identifying disseminated infectious foci, whereas a negative valvular PET/CT examination cannot reliably exclude native-valve endocarditis [1,11].

The role of cardiac CT may expand further through spectral imaging, photon-counting detector CT, deep learning reconstruction (DLR), quantitative image analysis, and multimodal artificial intelligence (AI). These technologies may improve spatial resolution, iodine conspicuity, artifact reduction, thin-section image quality, and computational characterization of valvular and periannular lesions [15,16,17,18,19,20]. However, most supporting evidence has been obtained from coronary imaging, prosthetic-valve phantoms, or broader cardiovascular applications rather than dedicated IE cohorts. Improved technical image quality should therefore not be equated with improved diagnostic accuracy or clinical outcomes without direct validation.

Previous publications have addressed multimodality imaging or the general role of cardiac CT in IE. Nevertheless, a radiologically oriented synthesis is needed to connect CT acquisition strategy with lesion-specific morphology, comparative interpretation with echocardiography, prosthetic and device-related infection, extracardiac assessment, surgical relevance, and emerging technologies. Accordingly, this Review critically examines the contemporary role of cardiac CT in IE and distinguishes established clinical applications from indirect technological evidence and conceptual future developments. Particular emphasis is placed on structural complications, substrate-specific applications, preoperative anatomical mapping, and the translational limitations of spectral CT, photon-counting CT, radiomics, and artificial intelligence.

## 2. Review Scope and Evidence Selection

This manuscript was conducted as a narrative review rather than a systematic review or meta-analysis. The literature search was designed to identify clinically and technically relevant publications addressing the role of cardiac computed tomography in infective endocarditis and related multimodality imaging applications. Relevant literature was identified through targeted searches of PubMed/MEDLINE up to August 2026, supplemented by citation tracking of international guidelines, expert consensus documents, landmark studies, and previous evidence syntheses.

The main search concepts included combinations of “infective endocarditis”, “cardiac computed tomography”, “cardiac CT”, “computed tomography angiography”, “transesophageal echocardiography”, “prosthetic valve endocarditis”, “cardiac implantable electronic device infection”, “periannular complications”, “abscess”, “pseudoaneurysm”, “FDG PET/CT”, “WBC-SPECT/CT”, “photon-counting CT”, “spectral CT”, “radiomics”, and “artificial intelligence”. Only English-language publications were considered. Priority was given to studies that evaluated cardiac CT in patients with suspected or confirmed infective endocarditis; compared CT with echocardiography, nuclear imaging, surgery, pathology, or multidisciplinary clinical diagnosis; or examined the anatomical and clinical significance of valvular, periannular, prosthetic, device-related, coronary, and extracardiac complications. Selected case reports and technical studies were included only when they illustrated uncommon manifestations or emerging imaging applications that were not adequately represented in larger clinical cohorts.

Accordingly, no PRISMA flow diagram, protocol registration, formal risk-of-bias assessment, or GRADE certainty-of-evidence evaluation was performed. Evidence was instead interpreted qualitatively according to study design, clinical relevance, methodological limitations, and the directness of its applicability to infective endocarditis.

Evidence was organized according to diagnostic criteria, cardiac CT acquisition and reconstruction, lesion-specific structural complications, infected clinical substrate, extracardiac and preoperative assessment, emerging technologies, and evidence gaps and future research priorities. Comparative observations regarding echocardiography and selected multimodality techniques were incorporated within the relevant lesion- and substrate-specific sections rather than treated as separate stand-alone topics. Diagnostic-performance estimates were derived from the cited primary studies and meta-analyses rather than recalculated. Because the available literature is heterogeneous with respect to patient selection, valve type, scanner technology, acquisition protocol, lesion definition, and reference standard, no new quantitative synthesis was performed.

Evidence regarding spectral CT, photon-counting detector CT, deep learning reconstruction, radiomics, and artificial intelligence was classified according to its level of direct applicability to infective endocarditis. A distinction was maintained among direct clinical evidence from IE populations, indirect evidence extrapolated from broader cardiovascular imaging, and conceptual applications that have not yet undergone IE-specific validation.

The clinical images included for illustrative purposes are original de-identified images obtained during routine clinical care at the authors’ institution and were not reproduced from previously published reports. Their use is described in the Institutional Review Board and Informed Consent statements below.

## 3. Contemporary Diagnostic Criteria and the Expanding Role of Imaging

### 3.1. Evolution from the Duke Criteria to Multimodality Imaging

The diagnosis of infective endocarditis requires integration of clinical, microbiological, imaging, surgical, and, when available, pathological evidence. Although microbiological or histopathological confirmation in excised tissue provides the most definitive evidence of infection, such information is frequently unavailable when therapeutic decisions must be made and may never be obtained in patients managed without surgery. Diagnostic criteria therefore provide a standardized framework for combining complementary findings, but they should not replace clinical judgment or multidisciplinary assessment.

The original Duke criteria, introduced in 1994, classified cases as definite, possible, or rejected IE by combining major microbiological and echocardiographic findings with minor clinical criteria [21]. The modified Duke criteria subsequently refined the definitions of typical microorganisms, positive blood cultures, echocardiographic findings, and the combinations required for diagnostic classification [22]. These criteria became the predominant diagnostic framework for clinical practice and research.

However, both systems were developed when echocardiography was effectively the only imaging modality incorporated into formal diagnostic classification. Their sensitivity is therefore reduced in patients with prosthetic valves, intracardiac devices, transcatheter valves, previous reconstructive surgery, or extensive periannular infection. In these settings, acoustic shadowing, reverberation, calcification, and surgically altered anatomy may obscure both vegetations and local complications, while prior antimicrobial therapy may reduce microbiological yield [4,5,11].

The 2015 ESC guidelines marked a transition toward multimodality imaging [8]. In addition to echocardiographic evidence of endocardial involvement, paravalvular lesions detected by cardiac CT were recognized as major imaging findings. Abnormal periprosthetic activity identified by ^18^F-FDG PET/CT or radiolabeled leukocyte SPECT/CT was also incorporated as major evidence under defined clinical conditions [8]. This development acknowledged that echocardiography, cardiac CT, and nuclear imaging depict different pathological components of IE: dynamic valve dysfunction, anatomical tissue destruction, and inflammatory or infectious activity.

### 3.2. The 2023 Duke-ISCVID and ESC Criteria

The 2023 Duke–International Society for Cardiovascular Infectious Diseases criteria retained the general structure of the modified Duke framework while expanding the recognized microbiological, molecular, imaging, pathological, and surgical evidence [6]. The update reflects major changes in contemporary IE, including the increasing prevalence of prosthetic and transcatheter valves, cardiac implantable electronic devices, healthcare-associated infection, culture-negative disease, and the availability of advanced microbiological and imaging techniques.

The updated major imaging criterion encompasses characteristic anatomical or metabolic evidence of valvular, periannular, periprosthetic, or device-related infection. Cardiac CT contributes particularly to the detection of abscesses, pseudoaneurysms, fistulous tracts, and prosthetic dehiscence, whereas metabolic imaging may provide additional evidence of active infection involving prosthetic valves and implanted cardiovascular material [6].

The 2023 ESC guidelines similarly emphasize early, substrate-specific multimodality imaging. Echocardiography remains the initial imaging approach, but cardiac CT, ^18^F-FDG PET/CT, PET/CTA, and radiolabeled leukocyte SPECT/CT are incorporated according to the infected substrate, the results of initial echocardiography, and the suspected complication [5]. The choice of modality is therefore driven by the unresolved diagnostic question rather than by a fixed sequence applied uniformly to every patient.

The 2023 framework also broadens the role of direct surgical evidence. This is clinically relevant because destructive periannular disease may be identified intraoperatively despite incomplete preoperative fulfillment of clinical criteria. Nevertheless, surgical evidence is unavailable in patients treated medically and cannot serve as a universal reference standard.

### 3.3. Validation and Limitations of the Contemporary Criteria

External validation studies indicate that the 2023 Duke-ISCVID criteria improve sensitivity relative to earlier diagnostic systems while generally preserving high specificity.

In a cohort of 595 consecutive patients referred for suspected IE, of whom 399 were adjudicated as having the disease, the 2023 Duke-ISCVID criteria achieved a sensitivity of 84.2%, compared with 74.9% for the modified Duke criteria and 80.0% for the 2015 ESC criteria [7]. The improved classification was driven primarily by revisions to the major microbiological and imaging criteria. Compared with the 2023 ESC framework, Duke-ISCVID demonstrated similar sensitivity but greater specificity in that cohort [7].

A separate validation study involving 661 episodes referred to the Lund Infective Endocarditis Team also found greater sensitivity for the Duke-ISCVID criteria than for the modified Duke criteria, although the absolute improvement was moderate. Sensitivity for definite IE was 72.5% with Duke-ISCVID, 70.7% with the 2015 ESC criteria, and 66.1% with the modified Duke criteria; specificity remained above 92% for all three systems [9].

These results support the contemporary criteria as improved diagnostic frameworks, but important limitations remain. A proportion of surgically confirmed cases still fail to meet the criteria for definite IE before surgery, demonstrating that incomplete classification does not exclude disease when clinical suspicion remains high [7]. Conversely, treating both possible and definite classifications as a positive diagnosis substantially increases sensitivity at the expense of specificity [9].

Diagnostic criteria should consequently be interpreted alongside:

1. Pretest probability;
2. Microbiological plausibility;
3. Imaging quality and timing;
4. Exposure to antimicrobial therapy;
5. Prosthesis or device type;
6. Evolution during follow-up;
7. Multidisciplinary assessment.

A classification of possible IE should prompt further evaluation rather than automatic confirmation, while a rejected or incomplete classification should not terminate investigation when imaging or microbiological findings remain strongly suggestive.

### 3.4. Practical Integration of Imaging Criteria

The inclusion of advanced imaging within diagnostic criteria does not imply that every available modality should be performed routinely. Imaging should follow a lesion-oriented and substrate-specific strategy.

TTE remains the first-line examination, with TEE used when clinical suspicion persists, TTE is negative or inconclusive, prosthetic or device-related infection is suspected, or local complications require further assessment [5,11]. Cardiac CT should be added when the unresolved diagnostic question is primarily anatomical, particularly in suspected periannular extension, prosthetic or transcatheter-valve involvement, complex postoperative anatomy, or preoperative mapping [10,12,13,14].

^18^F-FDG PET/CT is most established in prosthetic-valve and device-related infection and provides whole-body assessment for metastatic infection and potential portals of entry. Radiolabeled leukocyte SPECT/CT may offer greater specificity for active pyogenic infection when FDG uptake is equivocal or potentially related to sterile postoperative inflammation [5,23].

The optimal pathway therefore integrates:

1. Echocardiography for dynamic morphology, flow, and hemodynamics;
2. Cardiac CT for periannular anatomy and surgical mapping;
3. PET/CT or WBC-SPECT/CT for metabolic or cellular evidence of infection;
4. Microbiology and clinical findings for etiological and probabilistic assessment;
5. The Endocarditis Team for final diagnostic and management decisions.

**Figure 1:**
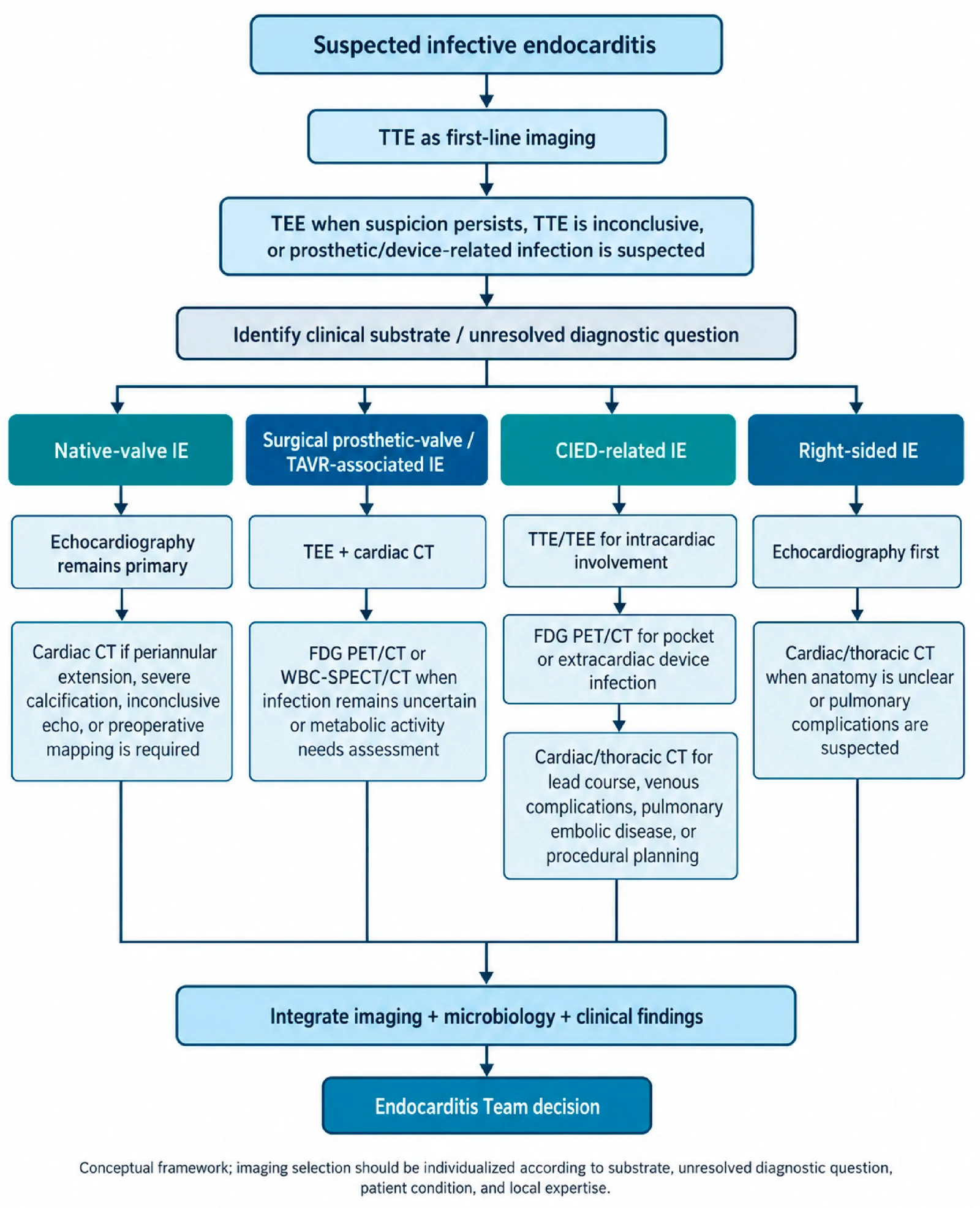
Proposed substrate-specific multimodality imaging pathway in suspected infective endocarditis. Echocardiography remains the first-line imaging approach, while cardiac CT and metabolic imaging are selected according to the infected substrate and unresolved diagnostic question. The pathway is intended as a conceptual framework and should be adapted to patient-specific factors, local expertise, and multidisciplinary assessment.

A practical substrate-specific multimodality imaging pathway integrating echocardiography, cardiac CT, and metabolic imaging is summarized in Figure 1. This flowchart is intended as a conceptual clinical framework rather than a rigid diagnostic algorithm, as imaging selection should remain guided by the infected substrate, the unresolved diagnostic question, local expertise, and the patient’s clinical condition.

## 4. Cardiac CT Acquisition and Reconstruction Protocols

### 4.1. Protocol Selection and Anatomical Coverage

Cardiac CT for suspected infective endocarditis should be tailored to the clinical question rather than performed as an unmodified coronary CT angiography (CTA) examination. Protocol selection depends on the suspected infected substrate, valve position, presence and type of prosthetic material, likelihood of periannular extension, need for coronary assessment, and possibility of extracardiac complications. A protocol optimized exclusively for coronary visualization may provide insufficient temporal coverage for evaluating valve motion, prosthetic instability, or dynamically changing lesions. Conversely, routine full-cycle and extended-body imaging may produce unnecessary radiation and contrast exposure when the diagnostic question is limited to a static periannular abnormality [12,24].

The core examination generally consists of a contrast-enhanced, ECG-synchronized cardiac acquisition with thin, near-isotropic sections covering the entire heart. A targeted non-enhanced acquisition may be added when calcification, prosthetic components, surgical pledgets, or other high-attenuation material must be distinguished from contrast enhancement. It should not be performed routinely when it is unlikely to influence interpretation because it increases radiation exposure [12,24].

Delayed thoracic or extracardiac imaging should be selected according to symptoms and the anticipated effect on management. Extended coverage may be appropriate when septic pulmonary emboli, systemic embolization, abscesses, infected aneurysms, spondylodiscitis, or other metastatic infectious complications are suspected. The anatomical range and number of phases should therefore be individualized rather than applied uniformly to every patient [5,12,24].

### 4.2. Patient Preparation and Contrast Administration

Patient preparation should account for cardiac rhythm, hemodynamic status, renal function, previous contrast exposure, venous access, respiratory cooperation, and the ability to tolerate breath holding. Patients with acute IE may have sepsis, severe regurgitation, heart failure, conduction abnormalities, hypotension, or renal dysfunction, and preparation protocols developed for elective coronary CTA may not always be appropriate.

Aggressive heart-rate reduction is not essential for every IE examination. Beta-blockers may be considered when simultaneous coronary artery evaluation is required and no contraindication is present, but they should not delay urgent imaging or compromise hemodynamic stability. Sublingual nitroglycerin may improve coronary visualization but is not required for valvular or periannular assessment and may be inappropriate in patients with hypotension or severe valvular dysfunction [13,24].

Contrast administration should be adapted to the anatomical side of suspected infection and the patient’s circulation. Conventional coronary CTA protocols usually provide satisfactory enhancement of the left atrium, left ventricle, aortic root, coronary arteries, and left-sided valves. Right-sided infection is more challenging because dense contrast in the superior vena cava and incomplete mixing within the right atrium may generate streak artifacts or pseudodefects. A diluted contrast–saline phase or another multiphasic injection strategy may improve homogeneous right-heart enhancement [24].

Abnormal cardiovascular physiology may also alter bolus arrival and distribution. Severe regurgitation, reduced cardiac output, intracardiac shunts, congenital heart disease, and surgically modified circulation may require individualized bolus timing. In the surgical cohort reported by Koo et al., 60–80 mL of iodinated contrast material was administered at 4 mL/s, followed by a contrast–saline mixture, with bolus tracking in the ascending aorta [13]. This protocol illustrates one combined valve and coronary strategy but should not be interpreted as a universal prescription.

### 4.3. ECG Synchronization and Multiphase Reconstruction

ECG synchronization is essential for reducing motion and accurately depicting the valves, annuli, prosthetic interfaces, and periannular tissues. A single prospectively triggered diastolic phase may be sufficient for selected static anatomical questions or isolated coronary assessment, but it is inadequate for comprehensive evaluation of dynamic valvular and prosthetic abnormalities.

Retrospective ECG gating or wide-window prospective triggering permits reconstruction throughout the cardiac cycle and is preferred when the examination must assess vegetation excursion, leaflet opening and closure, prosthetic rocking, dehiscence, fistulous communication, or dynamic filling of a pseudoaneurysm. Reconstructions at approximately 5–10% R–R intervals can produce cine datasets that demonstrate whether an abnormality is mobile, fixed, communicating with a cardiac chamber, or associated with abnormal prosthetic motion [10,12,13,24].

The importance of multiphase acquisition is supported by comparative evidence. In the meta-analysis by Oliveira et al., which included eight studies using surgical findings as the reference standard, CT demonstrated a pooled sensitivity of 78% (95% CI, 70–85%) and specificity of 92% (95% CI, 87–95%) for abscess or pseudoaneurysm, compared with 69% (95% CI, 62–76%) and 96% (95% CI, 94–98%), respectively, for TEE. When the analysis was restricted to the seven studies using multiphase CT, CT sensitivity increased to 87% (95% CI, 78–93%; 70/79 lesions) [10]. Multiphase CT also increased sensitivity for vegetation to 86% (95% CI, 80–91%) and for paravalvular leakage to 72% (95% CI, 51–88%) [10].

Full-cycle retrospective gating is associated with greater radiation exposure. Dose optimization should therefore include ECG-controlled tube-current modulation, appropriate tube-voltage selection, iterative or deep learning reconstruction, and restriction of the maximum-current window when clinically feasible. Wide-window prospective acquisition may provide a reasonable compromise, although its suitability depends on scanner technology, rhythm stability, heart rate, and the degree of functional information required.

Further radiation and contrast optimization may be achievable with prospective ECG-triggered high-pitch protocols in selected patients when comprehensive full-cycle functional information is not required. In a TAVI-planning population, a third-generation dual-source CT protocol using high-pitch acquisition provided diagnostic image quality with a mean effective radiation dose of 2.8 ± 0.3 mSv and 60 mL of iodinated contrast medium [25]. Although these data are not specific to infective endocarditis and should not be directly extrapolated to patients requiring multiphase assessment, they provide a useful technical framework for the development of lower-dose and lower-contrast cardiac CT protocols tailored to the clinical question.

Thin-section reconstruction, commonly in the range of approximately 0.60–0.75 mm with conventional multidetector CT, permits high-quality multiplanar reformations [12,13,24]. The smallest technically available section thickness should not automatically be selected, because excessive noise may offset the theoretical spatial-resolution benefit. Section thickness, reconstruction interval, kernel, matrix, and field of view should be chosen together according to scanner performance and the target structure.

### 4.4. Valve-Oriented Image Interpretation

Axial images alone are insufficient for evaluating infective endocarditis. Interpretation should use valve-aligned multiplanar reformations, double-oblique planes, and multiphase cine images. Three-dimensional volume-rendered images may improve communication with clinicians and surgeons, but all findings should be confirmed on thin-section source or multiplanar images because rendering thresholds may obscure small vegetations, narrow fistulous tracts, or low-attenuation periannular lesions.

The aortic valve should be assessed in a double-oblique short-axis plane, left ventricular outflow tract or three-chamber view, and planes parallel and perpendicular to the annulus and leaflets. Diastolic reconstructions are particularly useful for evaluating cusp coaptation, attached vegetations, the aortic root, and the mitral–aortic intervalvular fibrosa, whereas systolic images demonstrate maximal leaflet opening.

Mitral-valve assessment should include sagittal, coronal, three-chamber, four-chamber, and annular in-plane reconstructions. Systolic phases are often most informative for leaflet coaptation and mitral regurgitation-related structural abnormalities, while cine review may demonstrate prolapse, irregular leaflet motion, perforation, or prosthetic instability [13].

The selected cardiac phase and plane should be adapted to the lesion. A periannular collection may be characterized predominantly by high-resolution static images, whereas a suspected vegetation, dehiscence, fistula, or pseudoaneurysm requires evaluation over multiple phases. Koo et al. reconstructed datasets at 5% R–R intervals and used valve-specific double-oblique planes and cine imaging, achieving substantial-to-excellent interobserver agreement for the main CT findings [13].

### 4.5. Prosthetic Material, Artifact Reduction, and Advanced Reconstruction

Mechanical valves, annuloplasty rings, transcatheter frames, pacing leads, surgical clips, and other high-attenuation structures can produce blooming, beam-hardening, and streak artifacts. These artifacts may obscure small vegetations and periannular lesions or simulate contrast-filled gaps adjacent to prosthetic components [24,26].

Image quality can be improved through appropriate tube-voltage selection, thin-section reconstruction, optimized reconstruction kernels, iterative or deep learning reconstruction, dedicated metal-artifact-reduction techniques, and spectral reconstruction when available. High-keV virtual monoenergetic images may reduce metallic streaking and beam hardening, whereas lower-keV images increase iodine attenuation and may improve the conspicuity of enhancing tissue or contrast-filled channels. Because the optimal energy level depends on the prosthesis, patient habitus, and diagnostic target, several spectral reconstructions may need to be reviewed [24].

Photon-counting and ultra-high-resolution CT may reduce blooming and improve visualization around calcification or metallic structures. Mergen et al. demonstrated improved spatial resolution and reduced blooming in a coronary photon-counting CT cohort, while Boccalini et al. showed thinner apparent prosthetic components and fewer metallic artifacts in a prosthetic-valve phantom [27,28]. However, these studies do not establish improved diagnostic performance in IE, and simulated lesions of approximately 1.5–2 mm remained difficult to identify even with photon-counting CT [28].

Deep learning and super-resolution reconstruction may reduce noise and improve delineation of small or metallic structures. Studies by Tatsugami et al., Xu et al., and Orii et al. reported improvements in image noise, contrast-to-noise ratio, coronary sharpness, and visualization of stent lumens [18,19,20]. These findings are technically relevant but remain indirect because none of the studies evaluated the detection of IE-related lesions. Advanced reconstruction should therefore be regarded as an image-optimization strategy rather than an independently validated diagnostic method for endocarditis.

A lesion- and question-specific approach to acquisition and reconstruction is summarized in Table 1, allowing the protocol to be tailored to the suspected complication rather than applied uniformly to all patients.

## 5. Cardiac CT Appearance of Valvular and Periannular Structural Complications

### 5.1. Lesion-Oriented Interpretation

The structural manifestations of infective endocarditis represent a pathological continuum rather than a series of independent findings. Infection may initially involve a native leaflet, prosthetic component, endocardial surface, or intracardiac device and subsequently progress to leaflet destruction, periannular infiltration, abscess formation, pseudoaneurysm, fistulization, and prosthetic dehiscence. Cardiac CT interpretation should therefore be lesion-oriented while also assessing the complete anatomical pathway of infection [12,29].

A comprehensive examination should evaluate the valve or implanted material, annulus, surrounding fibrous and myocardial structures, adjacent cardiac chambers, coronary arteries, and previous surgical reconstruction. For each abnormality, the report should describe its location, dimensions, morphology, relationship to the annulus and prosthetic material, communication with the cardiovascular lumen, and potential implications for surgery. Particular attention should be given to extension toward the mitral–aortic intervalvular fibrosa, interventricular septum, coronary ostia, right ventricular outflow tract, atrial walls, and previous grafts or conduits.

**Figure 2:**
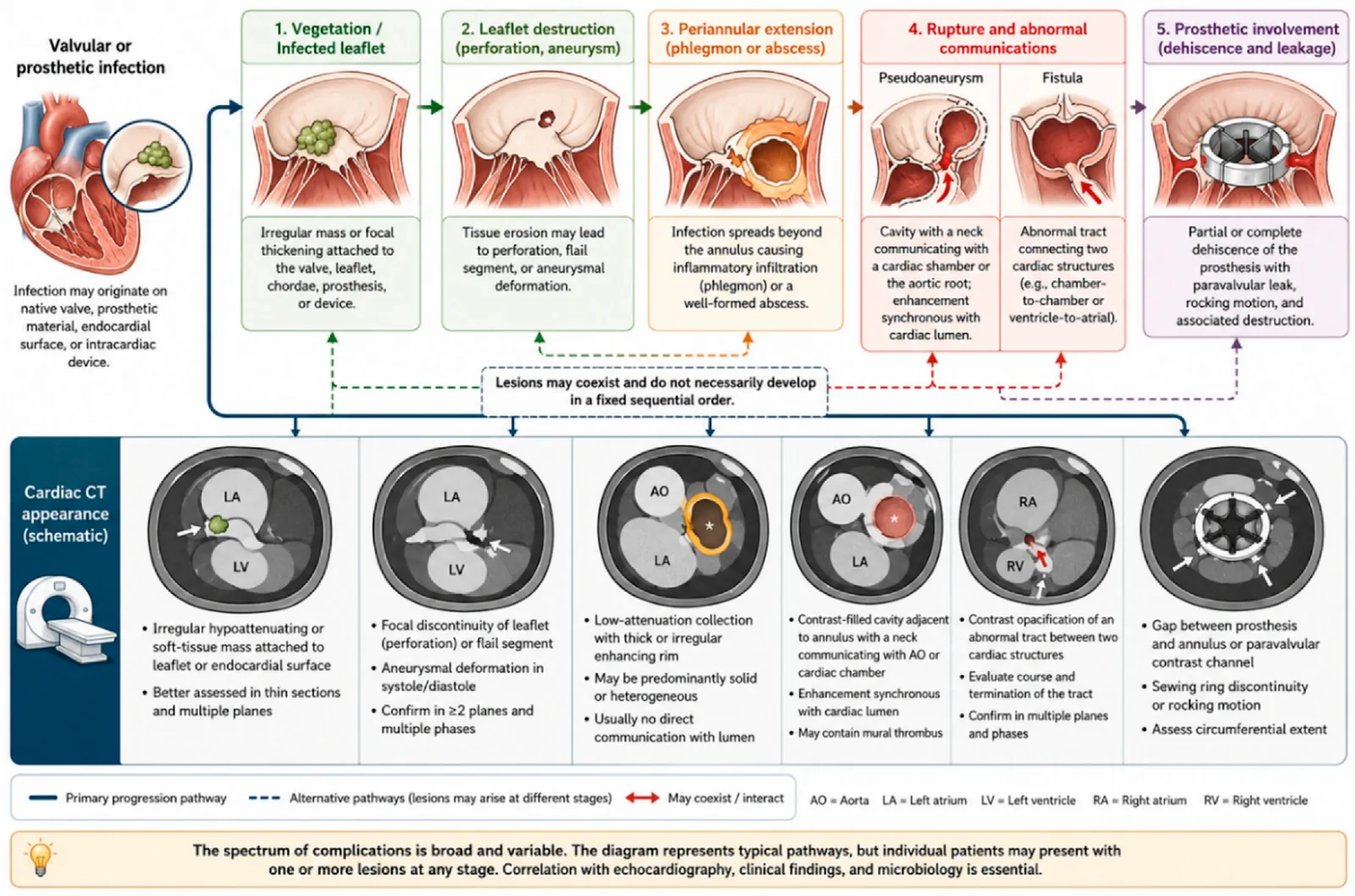
Structural progression of infective endocarditis and corresponding cardiac CT findings. Infection may progress from vegetation or infected leaflet thickening to tissue erosion, perforation, periannular inflammatory extension, abscess or pseudoaneurysm formation, fistulization, and prosthetic dehiscence. The sequence is schematic; individual lesions may coexist or develop through alternative pathways. Figure created by the authors.

Multiphase CT is important because several manifestations are dynamic. Vegetations may change position during the cardiac cycle, pseudoaneurysms may fill or expand, fistulous communications may become more conspicuous in selected phases, and prosthetic dehiscence may manifest as abnormal rocking motion [10,12,13,30]. The principal morphological progression and corresponding CT targets are summarized in Figure 2.

### 5.2. Vegetations and Leaflet Destruction

A vegetation is an infected mass attached to a valve leaflet, endocardial surface, chordal structure, prosthetic valve, intracardiac lead, or other implanted material. On cardiac CT, vegetations typically appear as irregular hypoattenuating or intermediate-attenuation soft-tissue masses attached to a valve or endocardial structure. In some patients, infection manifests only as focal or nodular leaflet thickening rather than a clearly defined mass [12,13,31].

The attachment site and relationship to the leaflet, annulus, prosthetic sewing ring, or subvalvular apparatus should be evaluated using valve-aligned multiplanar reformations and cine images. Although vegetations classically occur on the low-pressure side of the affected valve, their position may be variable in advanced infection or surgically altered anatomy. CT can additionally demonstrate associated calcification, tissue thickening, leaflet destruction, or extension beyond the valve.

**Figure 3:**
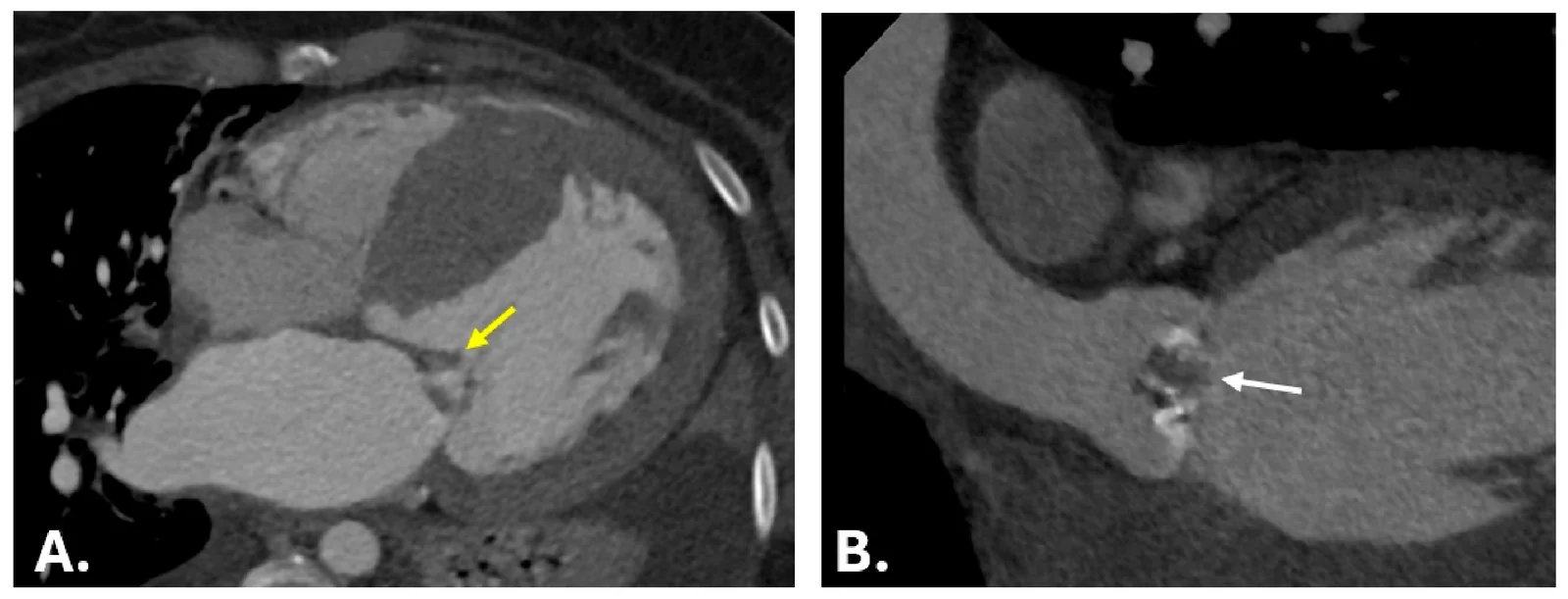
(**A**) Four-chamber contrast-enhanced cardiac CT shows multiple nodular hypoattenuating soft-tissue lesions attached to a native mitral valve (yellow arrow), representing valvular vegetations. (**B**) Oblique coronal coronary CT angiography shows a nodular hypoattenuating soft-tissue lesion (white arrow) attached to a native degenerated, calcified aortic valve.

Figure 3 shows two examples of vegetations on the native aortic valve and native mitral valve.

The principal limitation of CT is reduced sensitivity for small, thin, or highly mobile vegetations because temporal resolution remains lower than that of TEE. This limitation is especially relevant for lesions smaller than 10 mm and for lead-associated masses [10,31]. Consequently, the absence of a vegetation on CT does not exclude IE, and a negative examination should be interpreted in conjunction with TEE, microbiological findings, and clinical probability.

Potential mimics include thrombus, pannus, degenerative leaflet thickening, papillary fibroelastoma, Lambl excrescences, nonbacterial thrombotic endocarditis, and prosthesis-related artifacts. Morphology alone may not reliably distinguish these entities. A vegetation becomes more convincing when it is associated with destructive leaflet change, periannular inflammation, positive microbiology, or concordant mobility on echocardiography.

Progressive infection may cause focal erosion, tissue discontinuity, perforation, aneurysmal deformation, or extensive leaflet destruction. On CT, a perforation appears as a focal defect in the leaflet and should be confirmed in at least two perpendicular planes and across several cardiac phases to reduce false-positive interpretation caused by motion or oblique sectioning [13,31]. Multiphase imaging may demonstrate abnormal leaflet motion or contrast passage through the defect, but color Doppler TEE remains superior for identifying small perforations and evaluating the associated regurgitant jet [10,13].

A valvular aneurysm may appear as a focal saccular deformation or loss of the normal smooth leaflet contour that changes configuration during the cardiac cycle. Differentiation from an attached vegetation or motion artifact can be difficult, and echocardiographic correlation is generally required. The report should state whether there is focal thickening, definite tissue discontinuity, aneurysmal deformation, flail motion, or extensive destruction because the degree of tissue damage may influence the feasibility of valve repair.

### 5.3. Periannular Inflammatory Extension and Abscess

Periannular extension occurs when infection spreads from the valve or prosthetic sewing ring into adjacent fibrous tissue, myocardium, or the aortic root. It is especially important in aortic-valve and prosthetic-valve endocarditis because of the close relationship between the annulus, intervalvular fibrosa, conduction tissue, and coronary ostia.

Early inflammatory extension may appear as ill-defined periannular soft-tissue thickening, irregular enhancement, increased attenuation, loss of normal fat planes, or infiltration without a clearly formed cavity [12,14]. These findings may correspond to inflammatory infiltration or phlegmon. Their interpretation is challenging because postoperative fibrosis, chronic inflammation, and incomplete contrast mixing may produce similar appearances.

**Figure 4:**
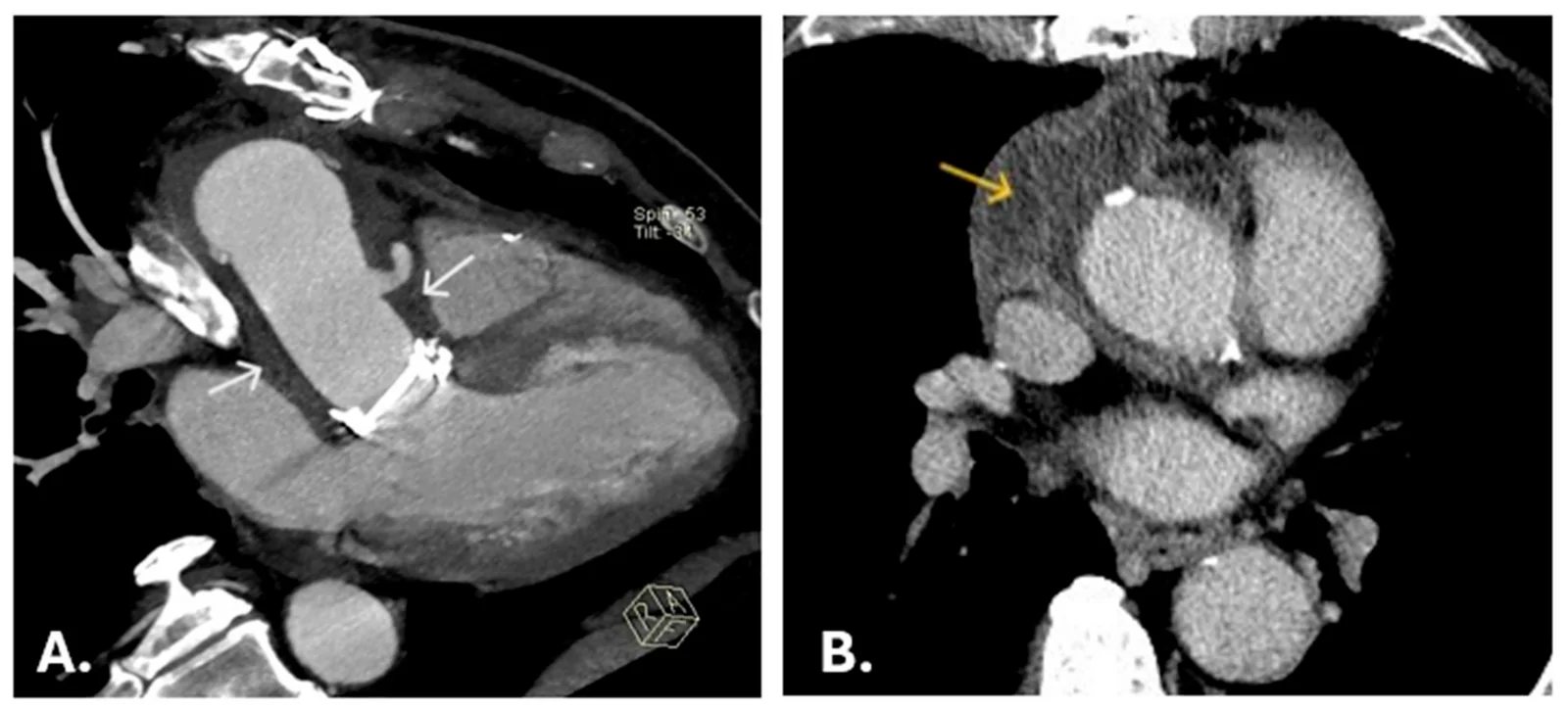
A 74-year-old male patient with mechanical aortic valve replacement. Three-chamber CTA image (**A**) shows a hypoattenuating fluid collection (white arrows) extending superiorly from the aortic root and surrounding the ascending aorta. Axial CT image in the venous phase (**B**) demonstrates a well-defined collection with enhancing walls, consistent with a periprosthetic abscess (yellow arrow). R, right; A, anterior; F, foot (inferior direction).

A mature abscess generally appears as a low- or fluid-attenuation periannular collection surrounded by thick, irregular, enhancing inflammatory tissue (Figure 4). Some abscesses are predominantly solid or heterogeneous rather than completely fluid, particularly after antimicrobial treatment or when partially organized. Unlike a pseudoaneurysm, an abscess does not necessarily communicate with a cardiac chamber or the aortic lumen and does not show synchronous luminal enhancement [12,13].

The distinction between phlegmon, abscess, and pseudoaneurysm is not always absolute. An abscess may rupture and evolve into a pseudoaneurysm, partial thrombosis may modify the enhancement pattern, and several abnormalities may coexist. Multiphase imaging and comparison with previous examinations can help determine whether a cavity communicates with the cardiovascular lumen and whether it changes during the cardiac cycle.

The value of CT lies not only in identifying the lesion but also in mapping its complete extent. The report should assess involvement of the aortic root, mitral–aortic intervalvular fibrosa, interventricular septum, coronary ostia, proximal coronary arteries, atrial walls, right ventricular outflow tract, pericardium, and previous surgical material. These relationships may determine the need for annular reconstruction, aortic-root replacement, intervalvular fibrosa reconstruction, coronary reimplantation, or additional debridement.

### 5.4. Pseudoaneurysms and Fistulous Communications

A pseudoaneurysm is a perivalvular cavity produced by infectious tissue destruction that communicates with a cardiac chamber or the aortic root. On CT, it appears as a contrast-filled cavity adjacent to the annulus or prosthetic valve, usually connected to the cardiovascular lumen through a visible or narrow neck. Enhancement synchronous with the adjacent chamber and dynamic change during the cardiac cycle distinguish a pseudoaneurysm from a noncommunicating abscess [12,13,31].

**Figure 5:**
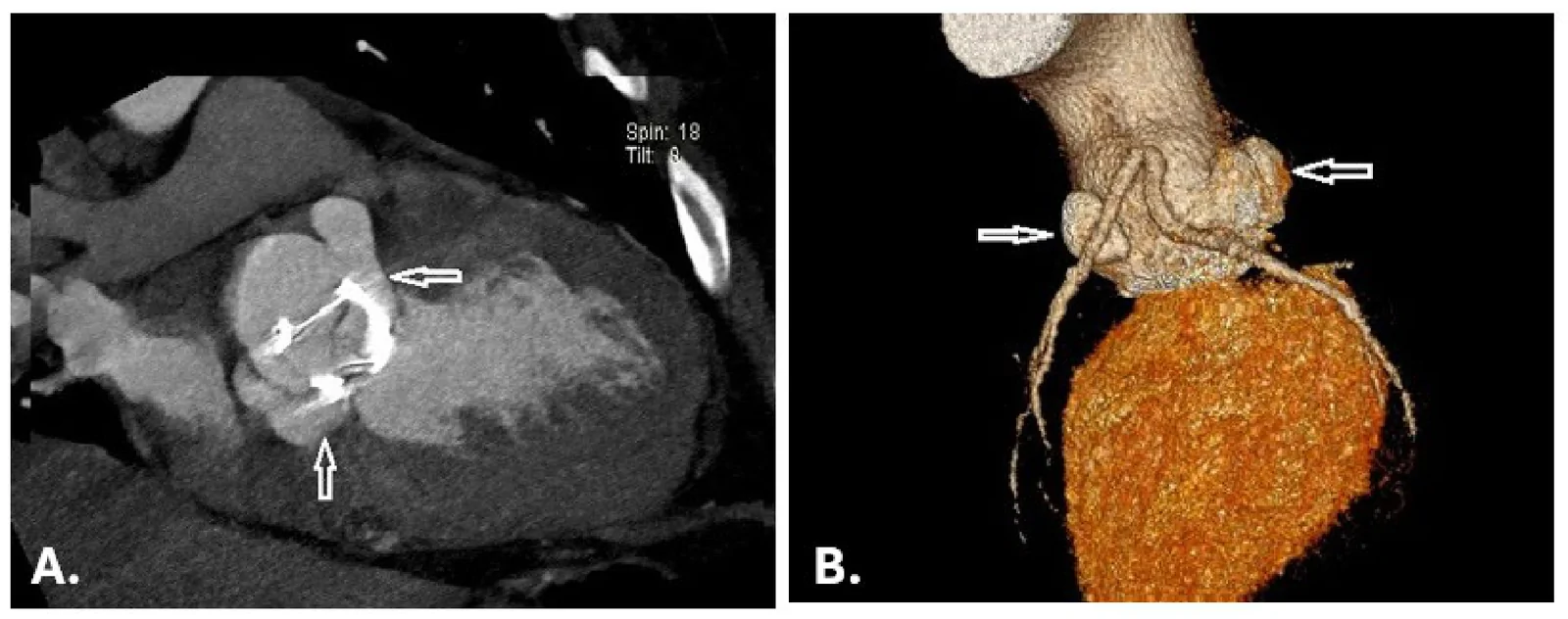
51-year-old male patient with infective endocarditis developed on an aortic bioprosthetic valve. Long-axis maximum intensity projection (MIP) CT angiography image (**A**) and 3D volume-rendering image (**B**) show two perivalvular cavities filled with contrast material (white arrows), representing two perivalvular pseudoaneurysms.

Pseudoaneurysms most frequently involve the aortic annulus, aortic root, or mitral–aortic intervalvular fibrosa. Multiplanar and multiphase reconstructions should be used to identify the communicating neck, define the chamber of origin, and establish the lesion’s relationship to the coronary arteries and surrounding structures (Figure 5). The report should document maximum dimensions, neck width, mural thrombus, associated inflammatory tissue, and evidence of fistulization or extracardiac extension.

Postoperative cavities, previous patch reconstruction, vascular grafts, coronary structures, sinus of Valsalva aneurysms, and chronic suture-line pseudoaneurysms may mimic active infection. Comparison with previous imaging is particularly important. Morphological stability, smooth margins, and established wall calcification may support a chronic postoperative abnormality, whereas new enlargement, irregular inflammatory tissue, or associated destructive change increases concern for active infection.

A fistula represents an abnormal communication between adjacent cardiovascular structures and usually reflects advanced destructive disease. On CT, it may appear as a continuous contrast-filled tract, focal tissue discontinuity, or abnormal opacification of a chamber or cavity through an unexpected route [12,30,31]. CT is particularly valuable for defining the origin, course, and termination of the tract, whereas TEE and color Doppler remain more sensitive for demonstrating active flow through small communications.

A suspected fistula should be visible in at least two planes and over multiple phases. Adjacent coronary arteries, prosthetic artifacts, partial-volume effects, and normal postoperative channels should be excluded before the finding is considered definite. Because fistulae frequently coexist with abscesses or pseudoaneurysms, the complete surrounding anatomy should be described rather than reporting the tract in isolation.

### 5.5. Prosthetic Dehiscence and Paravalvular Leakage

Prosthetic dehiscence results from disruption of the sewing ring, annulus, or surrounding tissue, producing partial separation of the prosthesis from its attachment site. On cardiac CT, dehiscence may manifest as a gap between the prosthesis and annulus, a contrast-filled paravalvular channel, discontinuity of the sewing ring, prosthetic malalignment, or abnormal rocking motion on cine images [13,30,31].

Cardiac CT is particularly useful for determining the circumferential extent of sewing-ring involvement and identifying associated abscess, pseudoaneurysm, or fistulous extension. Double-oblique reconstructions aligned with the prosthetic ring are essential because axial images may underestimate or mislocalize the defect. Abnormal motion should be interpreted according to the type and orientation of the prosthesis and should not be diagnosed solely from a single reconstruction.

TEE remains superior for demonstrating and quantifying paravalvular regurgitant flow. CT instead provides the structural correlate, including the location and extent of the gap, relationship to the annulus, and associated periannular destruction. The two modalities therefore answer different but complementary questions.

Important mimics include chronic noninfectious paravalvular leakage, normal postoperative gaps, suture pledgets, metallic blooming, beam-hardening artifacts, pannus, and thrombus. Suture pledgets usually appear as regularly distributed hyperattenuating structures around the sewing ring and remain visible on non-enhanced images. Comparison with previous examinations and integration with metabolic imaging may be required when the morphology is indeterminate.

### 5.6. Lesion-Specific Multimodality Interpretation

No imaging modality provides optimal assessment of every structural manifestation. TEE is generally more sensitive for small vegetations, leaflet perforations, regurgitation, and dynamic intracardiac flow. Cardiac CT provides superior or complementary anatomical assessment of periannular extension, abscesses, pseudoaneurysms, circumferential prosthetic involvement, coronary relationships, and surgically altered anatomy [10,12,13,14,31,32].

The practical objective is therefore not to identify a universally superior modality but to select the technique that best answers the unresolved lesion-specific question. TEE and multiphase cardiac CT should be interpreted together when there is suspected periannular extension, prosthetic-valve infection, discordance between clinical and echocardiographic findings, or a need for detailed preoperative mapping. The lesion-specific CT appearances, interpretive limitations, and complementary imaging roles are summarized in Table 2.

The principal head-to-head studies and meta-analyses supporting these lesion-specific differences are summarized in Table 3.

## 6. Cardiac CT Across Clinical Substrates

### 6.1. Native-Valve Endocarditis

Echocardiography remains the primary imaging approach in native-valve endocarditis because of its temporal resolution and ability to demonstrate vegetation mobility, leaflet perforation, regurgitation, and hemodynamic consequences. TTE is generally performed first, followed by TEE when the initial examination is negative or inconclusive, clinical suspicion remains high, or local complications are suspected [5,11,32].

The incremental value of cardiac CT is limited when high-quality echocardiography clearly demonstrates uncomplicated native-valve infection without evidence of periannular extension. CT becomes most relevant when echocardiographic findings are inconclusive, the valve is severely calcified, periannular disease is suspected, or detailed preoperative and coronary anatomical assessment is required [10,12,13,24,29].

In aortic-valve endocarditis, CT should evaluate the annulus, aortic root, sinuses of Valsalva, mitral–aortic intervalvular fibrosa, interventricular septum, coronary ostia, and proximal coronary arteries. Aortic-valve infection is particularly prone to periannular extension because of the close relationship between the annulus and the fibrous cardiac skeleton [12,29].

**Figure 6:**
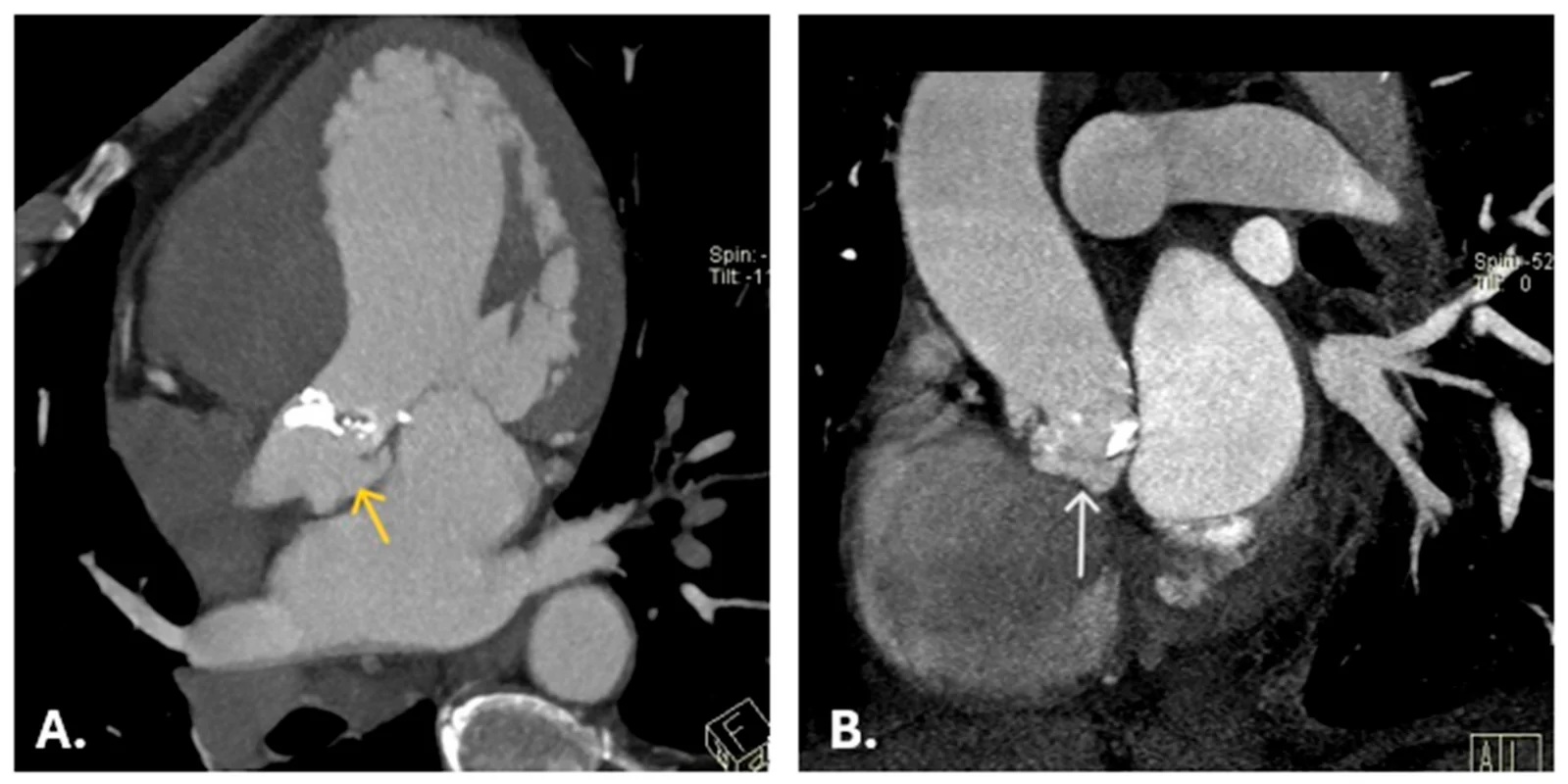
(**A**) Maximum-intensity projection (MIP) image of coronary CT angiography shows a perivalvular cavity filled with contrast material (yellow arrow) adjacent to a native degenerated, calcified aortic valve, representing a pseudoaneurysm. (**B**) Coronary CT angiography shows the perivalvular pseudoaneurysm (white arrow) communicating with the aortic root, consistent with an aortic root/aortic annulus pseudoaneurysm related to aortic-valve endocarditis.

Figure 6 illustrates two examples of pseudoaneurysms associated with native aortic valve infective endocarditis.

In mitral-valve endocarditis, CT may characterize leaflet destruction, annular involvement, extension into the intervalvular fibrosa, and the relationship of infection to the circumflex coronary artery. Nevertheless, small perforations and regurgitant jets remain more accurately assessed using TEE and color Doppler [10,13,30].

A negative CT examination cannot exclude native-valve endocarditis, particularly when vegetations are small, thin, or highly mobile. Its principal role is therefore to characterize the anatomical consequences of infection rather than to replace echocardiography for primary vegetation detection.

### 6.2. Surgical Prosthetic-Valve Endocarditis

Prosthetic-valve endocarditis represents one of the strongest indications for early multimodality imaging. Acoustic shadowing and reverberation from the prosthetic ring, leaflets, discs, or supporting structures may obscure vegetations and periannular complications. Although TEE remains the first-line examination, its diagnostic sensitivity is reduced compared with that in uncomplicated native-valve disease [11,32].

Cardiac CT provides unrestricted multiplanar visualization of the prosthetic valve, sewing ring, annulus, aortic root, and surrounding structures. Its greatest contribution lies in the detection and mapping of periannular inflammatory extension, abscesses, pseudoaneurysms, fistulae, sewing-ring disruption, and prosthetic dehiscence. Multiphase imaging can additionally demonstrate abnormal prosthetic motion or rocking [12,24,29,32].

**Figure 7:**
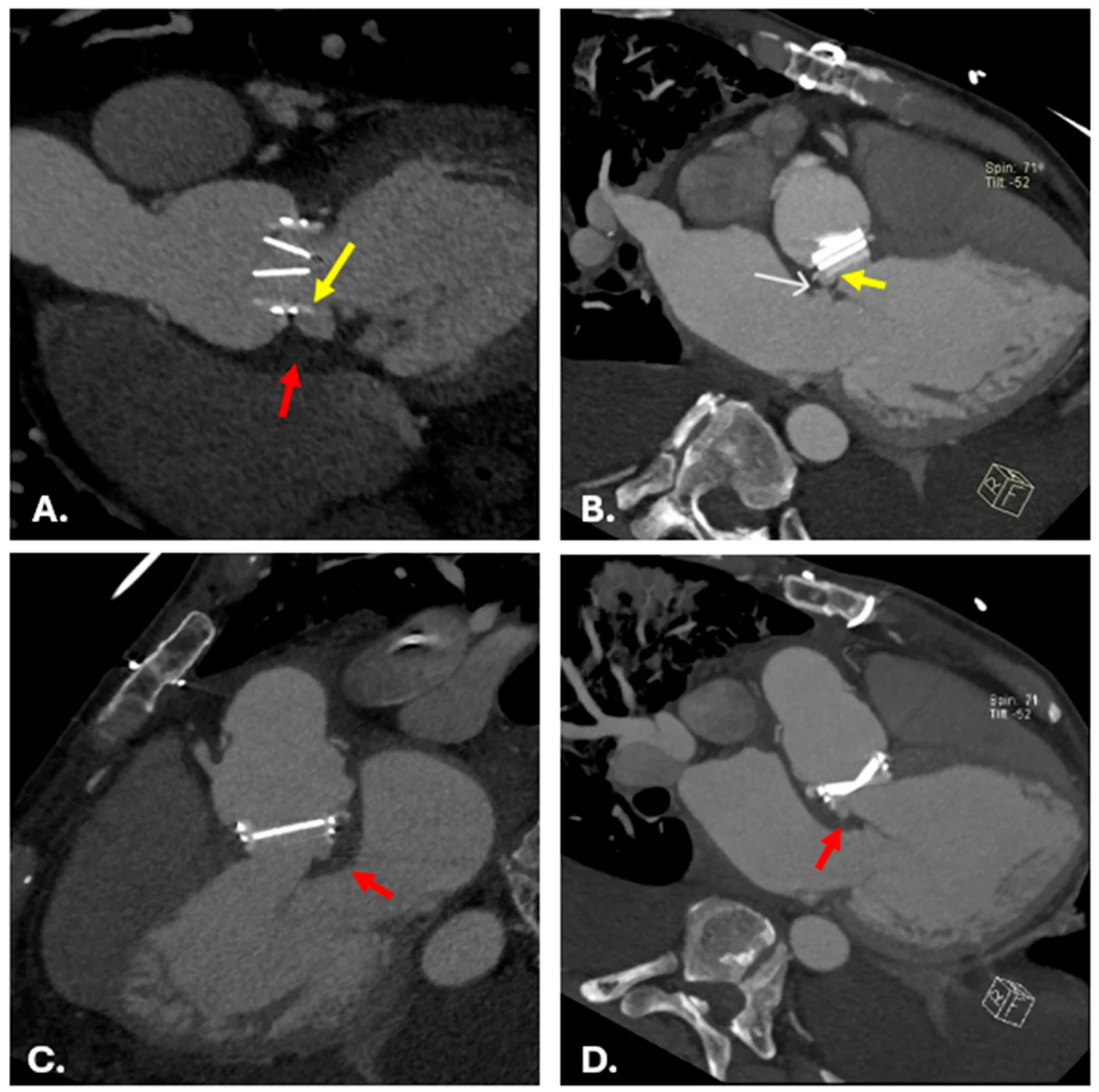
Coronary CT angiography of a 61-year-old male patient with infective endocarditis developed on a mechanical aortic valve. Images (**A**,**B**) show a pseudoaneurysm (yellow arrow) which extends towards the interatrial septum and left atrium, suggestive of a coexisting fistula (white arrow). Red arrow (images (**A**,**C**,**D**)) indicates a periprosthetic hypoattenuating fluid collection, representing a periprosthetic abscess, surrounding the left ventricular outflow tract (LVOT) and extending to the mitral–aortic intervalvular fibrosa (image (**D**)).

**Figure 8:**
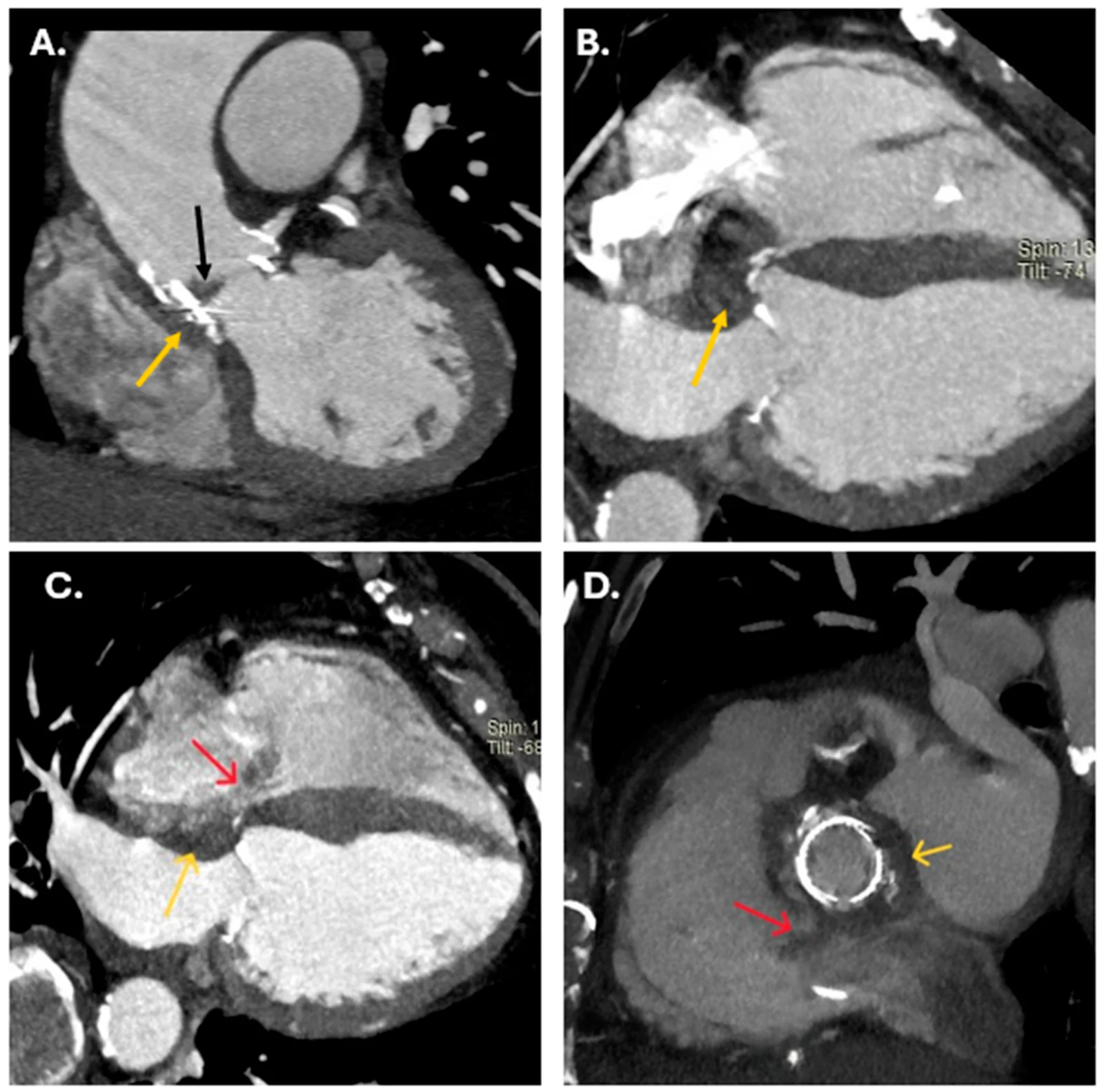
Coronary CT angiography of a 68-year-old female patient with infective endocarditis of a bioprosthetic aortic valve. (**A**) Nodular hypoattenuating soft-tissue lesion attached to the bioprosthetic valve (black arrow), consistent with a vegetation. (**B**–**D**) Yellow arrows indicate a periprosthetic hypoattenuating fluid collection, consistent with a periprosthetic abscess. (**C**,**D**) Red arrows indicate fistulous extension toward the tricuspid valve. Panel (**C**) shows a four-chamber view, and panel (**D**) a short-axis view. Beyond diagnosis, CT can define the circumferential extent of sewing-ring involvement and the relationship of infection to the coronary ostia, bypass grafts, previous aortic reconstruction, and structures relevant to repeat sternotomy. These findings may directly influence the required extent of debridement and reconstruction.

Figure 7 and Figure 8 illustrate two cases of prosthetic-valve infective endocarditis associated with perivalvular complications.

Interpretation must account for substantial morphological overlap between infection and noninfectious prosthetic dysfunction. Thrombus, pannus, calcific degeneration, postoperative fibrosis, suture pledgets, and chronic stable paravalvular leakage may mimic active infection. Comparison with previous examinations is therefore essential. CT attenuation may provide supportive information when differentiating pannus from thrombus. Pannus generally demonstrates higher attenuation than thrombus, and a threshold of approximately 145 HU has been proposed for their differentiation [29]. However, attenuation values overlap among thrombus, vegetation, infected tissue, and postoperative changes, and no validated Hounsfield-unit threshold establishes the presence of infection. Attenuation measurements should therefore not be interpreted in isolation but in conjunction with lesion morphology, enhancement, location, surrounding structural changes, and the clinical and microbiological context.

Suture pledgets are a particularly important pitfall. They may appear as regularly distributed hyperattenuating structures around the sewing ring and can simulate paravalvular contrast leakage. Their visibility on non-enhanced images, symmetrical arrangement, and stability over time support a postoperative rather than infectious origin [32].

### 6.3. Transcatheter-Valve Endocarditis

Infective endocarditis after transcatheter aortic valve implantation (TAVI) presents specific diagnostic challenges because of the metallic frame, heavily calcified native leaflets, altered aortic-root anatomy, and overlap between infection, leaflet thrombosis, structural degeneration, and baseline paravalvular regurgitation.

The SwissTAVI registry demonstrated that the risk of IE is greatest during the early period after implantation and that infection is associated with substantial mortality and stroke risk [33]. However, epidemiological detail should not dominate this radiological review; its relevance lies in emphasizing that transcatheter-valve infection is an increasingly important prosthetic substrate.

TEE may be impaired by shadowing from the transcatheter frame and retained calcified tissue. Cardiac CT can evaluate prosthesis position, frame expansion, leaflet morphology, annular interface, periannular extension, abscess, pseudoaneurysm, fistula, and paravalvular defects [24,29,32].

Hypoattenuating leaflet thickening is not specific for infection and is frequently associated with transcatheter-valve thrombosis. Infection should therefore be considered more likely when leaflet abnormalities coexist with periannular inflammation, tissue destruction, a new paravalvular defect, positive microbiology, or focal metabolic activity on FDG PET/CT.

The combination of anatomical CT and metabolic imaging is particularly useful when echocardiography is equivocal. CT localizes structural abnormalities in relation to the frame, leaflets, annulus, and aortic root, while PET/CT may demonstrate active inflammation involving the prosthetic interface.

Detailed discussion of ultra-high-resolution and photon-counting CT is provided in Section 8. Their inclusion here is limited to acknowledging that artifact reduction may be useful around metallic and calcified structures, while IE-specific clinical validation remains insufficient.

### 6.4. Cardiac Implantable Electronic Device (CIED)-Related Infection

CIED infection may involve the generator pocket, extracardiac lead segments, intravascular leads, cardiac valves, or several components simultaneously and should be considered an important contemporary substrate of infective endocarditis rather than solely an imaging scenario. Clinically, it is useful to distinguish isolated pocket infection, systemic CIED infection, lead-associated infective endocarditis, and concomitant valvular involvement. Isolated pocket infection is confined to the generator pocket and local hardware, whereas systemic infection may occur with or without pocket manifestations and with or without visible vegetations. Lead-associated infective endocarditis involves the intravascular device components, while concomitant valvular involvement most frequently affects the tricuspid valve but may coexist with left-sided IE. Precise localization is clinically important because prognosis, antimicrobial management, extraction planning, and the timing and strategy of reimplantation differ according to the extent of infection [32].

**Figure 9:**
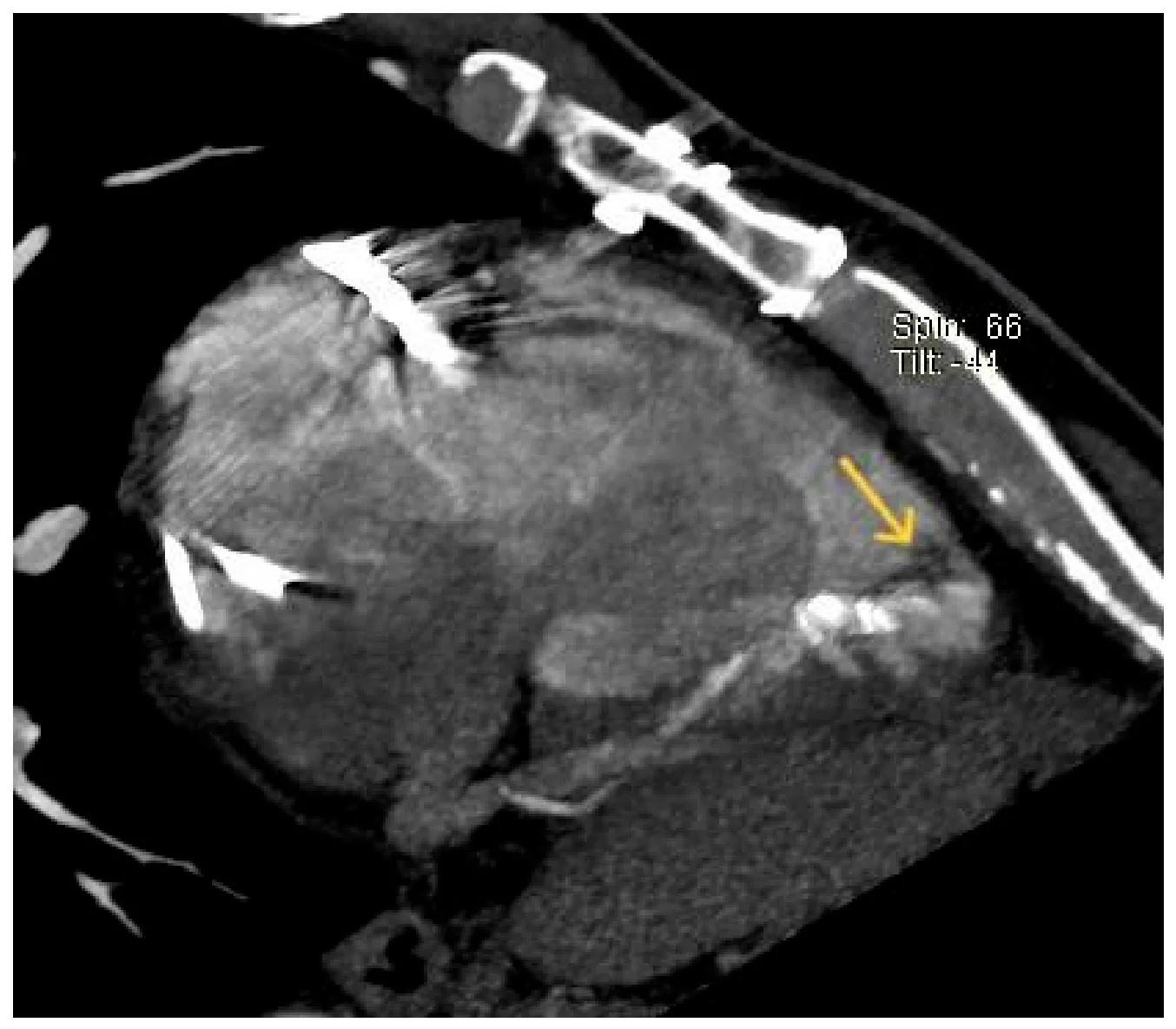
Oblique cardiac CT image showing a nodular hypoattenuating soft-tissue mass (yellow arrow) adjacent to and attached to a right ventricular lead. The hyperattenuating linear structure represents the intracardiac lead. The finding is consistent with a lead-associated mass.

TTE and TEE remain the initial imaging examinations when intracardiac lead or valvular involvement is suspected. TEE is more sensitive than TTE for lead-associated masses and tricuspid-valve involvement, although neither modality can reliably distinguish infected vegetation from sterile thrombus or fibrinous material in every case. Cardiac CT provides complementary anatomical information by mapping the intracardiac lead course, identifying larger lead-associated masses (Figure 9), assessing tricuspid-valve involvement, demonstrating venous obstruction or thrombosis, evaluating lead perforation, and detecting pulmonary septic emboli or other thoracic complications. Its principal limitations remain metallic artifacts, cardiac motion, and reduced sensitivity for small lead-associated lesions [24,32].

A mass identified on a lead should not automatically be classified as infected because chronic noninfectious lead-associated thrombi may occur. Interpretation requires integration with blood cultures, echocardiographic findings, generator-pocket abnormalities, metabolic imaging, and the clinical course. ^18^F-FDG PET/CT is particularly useful for assessing generator-pocket infection and extracardiac portions of the device, whereas cardiac CT provides more detailed anatomical characterization of structural, venous, and thoracic complications [23,32].

Once definite CIED-related infective endocarditis or confirmed lead infection is established, management extends beyond diagnostic imaging. Current European recommendations support complete removal of the CIED system in confirmed lead infection, together with appropriate antimicrobial therapy, because retention of infected hardware is associated with poorer outcomes [5]. Extraction should be performed in experienced centers, and the indication for subsequent device reimplantation should be reassessed rather than assumed. In patients with valvular infective endocarditis without definite lead involvement, the decision regarding CIED extraction should consider the causative microorganism, procedural risk, and the need for valve surgery [5].

Prevention requires assessment of patient-, procedure-, and device-related risk factors, treatment of active infection before implantation whenever feasible, appropriate peri-procedural antimicrobial prophylaxis, meticulous sterile technique, and strategies to minimize pocket hematoma and unnecessary reintervention [34]. Adjunctive local preventive approaches have also been investigated. A 10-year propensity-score-matched analysis evaluated the use of gentamicin-impregnated collagen sponges during CIED procedures; such approaches should be considered adjunctive to established preventive measures rather than substitutes for them [35].

The post-extraction period also requires individualized planning. Reimplantation should generally be deferred until clinical signs of infection have resolved and blood cultures have remained negative for the recommended interval, and a new system should be positioned away from the previously infected site [5]. In patients at high transient risk of sudden cardiac death after extraction of an infected implantable cardioverter–defibrillator, a wearable cardioverter–defibrillator may provide temporary protection while antimicrobial therapy is completed and the indication for permanent reimplantation is reassessed [5,36]. Multiparametric reassessment may further support individualized decisions regarding the continuing need for permanent defibrillator therapy [37]. Pacemaker-dependent patients may require temporary pacing while definitive reimplantation is deferred [5].

### 6.5. Right-Sided Endocarditis

Right-sided endocarditis most frequently involves the tricuspid valve and is associated with intracardiac devices, venous catheters, intravenous drug use, congenital heart disease, and previous cardiac procedures. Echocardiography remains the primary method for vegetation detection and assessment of tricuspid regurgitation.

Cardiac CT may provide incremental value when the relationship between a vegetation, valve, and implanted lead is unclear, when the pulmonary valve or right ventricular outflow tract is poorly visualized, or when congenital or postoperative anatomy is present. It also permits simultaneous evaluation of the lungs and pulmonary arteries for septic embolic complications [24,29].

The contrast-injection strategy must provide homogeneous enhancement of the right atrium and ventricle. Dense contrast in the superior vena cava and incomplete contrast–blood mixing may produce streak artifacts or pseudodefects. A diluted contrast–saline phase or tailored multiphasic injection may improve interpretation.

The thoracic acquisition should be reviewed for peripheral nodules, cavitation, wedge-shaped infarcts, pulmonary arterial filling defects, abscesses, pleural effusion, and empyema. These findings may support right-sided or device-related infection but are not specific and must be correlated with microbiology and intracardiac imaging.

### 6.6. Substrate-Specific Imaging Strategy

The contribution of cardiac CT varies according to the infected substrate. In native-valve IE, its main role is evaluation of periannular extension and preoperative anatomy; in surgical or transcatheter prosthetic-valve IE, it provides detailed assessment of the annular interface and surrounding tissues. In CIED-related infection, CT is primarily an anatomical and thoracic mapping tool, while active infection requires integration with echocardiography, microbiology, and metabolic imaging. In right-sided IE, cardiac assessment should be combined with evaluation of the pulmonary circulation and lungs.

Protocol design should therefore be individualized according to valve or device type, anatomical location, prosthetic material, suspected complication, and the complementary information already provided by echocardiography or nuclear imaging. The substrate-specific role of cardiac CT across native-valve, prosthetic-valve, transcatheter-valve, device-related, and right-sided infection is summarized in Table 4.

## 7. Extracardiac Complications and Preoperative Assessment

### 7.1. Multisystem Disease and CT Strategy

Infective endocarditis is a multisystem disease in which persistent bacteremia, embolization of infected material, and metastatic seeding may produce neurological, pulmonary, abdominal, vascular, musculoskeletal, and other extracardiac complications. These manifestations may precede recognition of the valvular infection, provide additional diagnostic evidence, influence the timing of surgery, or require treatment independently of the primary cardiac lesion [5,11,24,29].

Cardiac CT should therefore not be interpreted as an isolated examination of the valves. Even when anatomical coverage is restricted to the thorax, the lungs, pulmonary arteries, mediastinum, thoracic aorta, chest wall, skeletal structures, and visible upper abdominal organs should be reviewed systematically. Extended coverage should be added only when symptoms, microbiological findings, persistent infection, right-sided disease, or the planned intervention make extracardiac assessment clinically relevant.

A practical protocol may combine an ECG-synchronized cardiac acquisition with a delayed thoracic or thoracoabdominal phase adapted to the suspected complication. ECG synchronization is generally unnecessary outside the heart. The objective is not routine whole-body multiphase imaging in every patient, but selection of the smallest number of acquisitions capable of answering the relevant cardiac, vascular, and extracardiac questions [5,24].

### 7.2. Neurological and Systemic Embolic Complications

Neurological complications are among the most important extracardiac manifestations of IE because they can substantially influence prognosis and the timing of valve surgery. The spectrum includes ischemic infarction, septic emboli, intracranial hemorrhage, hemorrhagic transformation, cerebral abscess, meningitis, and infectious intracranial aneurysm [5,24,29].

Non-enhanced head CT remains appropriate for rapid evaluation of acute neurological deterioration, focal deficits, or suspected intracranial hemorrhage. Its availability and speed are advantageous in unstable patients, but a normal CT examination does not exclude small, early, or multifocal embolic lesions. Brain MRI is more sensitive for small ischemic foci, microhemorrhages, cerebral abscesses, and clinically silent embolization and should be preferred when the patient is stable and detailed neurological characterization is required [11].

CT angiography may be used when an infectious intracranial aneurysm is suspected, particularly in the presence of unexplained hemorrhage, focal neurological findings, recurrent embolization, or suspicious abnormalities on initial brain imaging. Very small or distal aneurysms may remain occult on CTA, and catheter angiography may still be required when clinical suspicion is high or endovascular treatment is being considered.

**Figure 10:**
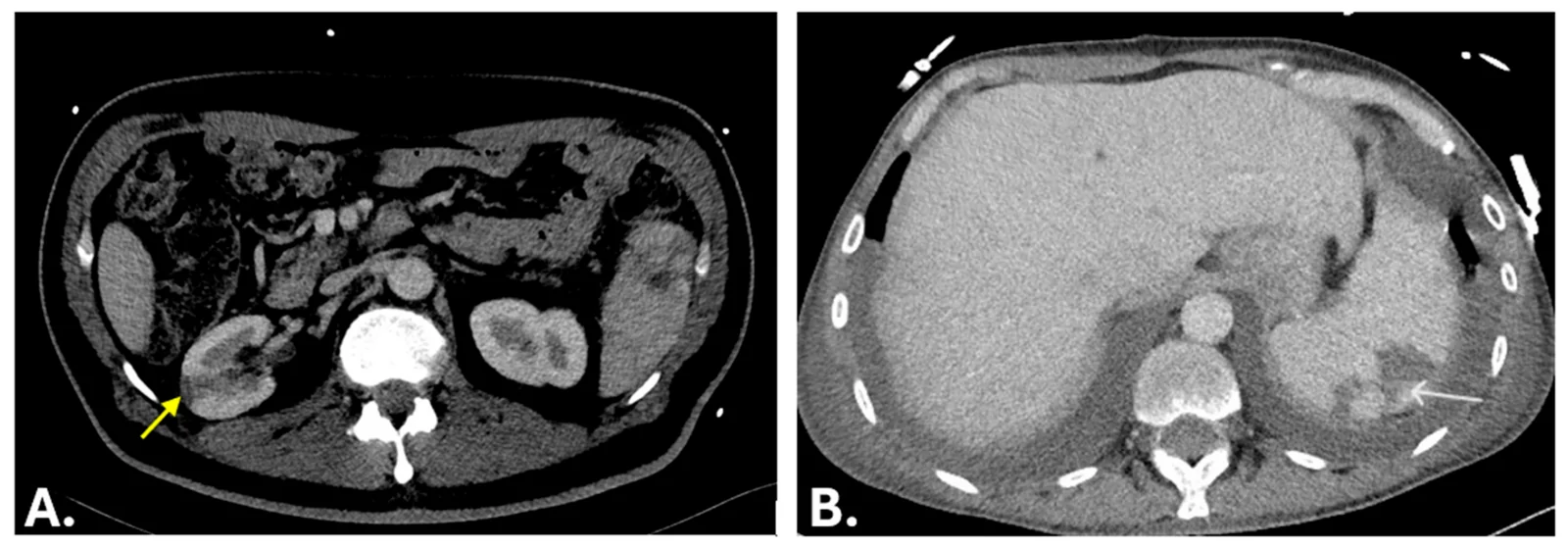
A 58-year-old male patient with infective endocarditis. Axial contrast-enhanced abdominal CT shows peripheral, wedge-shaped hypoattenuating regions in the right kidney (yellow arrow, (**A**)) and in the spleen (white arrow, (**B**)), consistent with renal and spleen infarcts.

Systemic embolization frequently involves spleen and kidneys. Acute infarcts usually appear as peripheral wedge-shaped or polygonal hypoattenuating regions (Figure 10). Secondary infection should be suspected when a lesion enlarges, becomes liquefied, develops peripheral enhancement or gas, or is associated with persistent bacteremia and local symptoms [24,29].

Splenic abscess, splenic rupture, renal abscess, mesenteric ischemia, or bowel infarction may alter the timing and sequence of cardiac and extracardiac interventions. By contrast, a small, uncomplicated infarct may provide supportive evidence of embolization without independently changing management. Imaging findings must therefore be interpreted according to their clinical consequences rather than merely counted as additional lesions.

### 7.3. Pulmonary and Osteoarticular Infection

**Figure 11:**
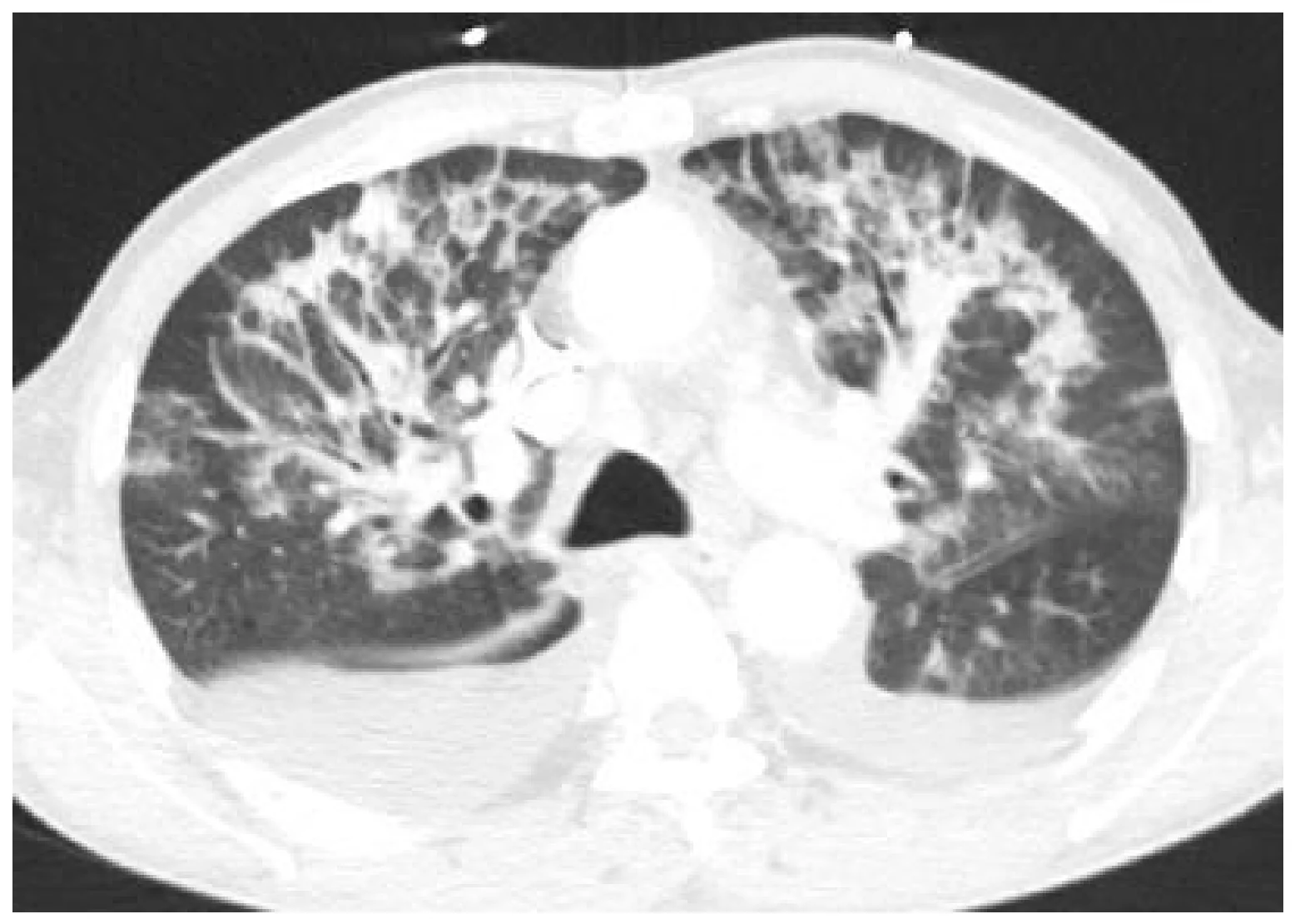
A 67-year-old male patient with infective endocarditis. Axial chest CT shows multiple foci of consolidation in both lungs, consistent with bronchopneumonia due to septic pulmonary emboli in the setting of infective endocarditis.

Pulmonary septic emboli are most strongly associated with tricuspid-valve endocarditis, infected intracardiac leads, central venous catheter infection, and other right-sided sources. CT manifestations include multiple pulmonary consolidations (Figure 11), cavitation, wedge-shaped pulmonary opacities, pulmonary arterial filling defects, abscess formation, pleural effusion, and empyema [24,29].

When right-sided or device-related IE is suspected, thoracic acquisition should include the complete lungs and pulmonary arteries. Pulmonary findings may support the diagnosis and influence antimicrobial, drainage, device-extraction, or surgical strategies. However, peripheral nodules and cavitation are not specific for IE and overlap with bland pulmonary infarction, fungal infection, metastatic disease, and inflammatory disorders.

Persistent back pain, focal spinal tenderness, joint symptoms, or unexplained bacteremia should raise suspicion for spondylodiscitis or another osteoarticular focus. CT may demonstrate endplate destruction, disc-space narrowing, paravertebral inflammatory tissue, or a psoas collection, but MRI remains more sensitive for early disc, marrow, epidural, and neural involvement [5,24]. The value of CT lies principally in detecting advanced osseous disease, identifying incidental abnormalities within extended coverage, and guiding biopsy or drainage.

### 7.4. Vascular, Coronary, and Nonvalvular Cardiac Complications

Infectious arterial aneurysms may develop through septic embolization, direct extension, infection of a pre-existing aneurysm, or involvement of vascular graft material. On CTA, suspicious features include a new irregular or saccular outpouching, rapid interval enlargement, surrounding inflammatory soft tissue, mural thickening, adjacent fluid, thrombosis, or contained rupture [24,29].

These lesions may involve intracranial, aortic, visceral, peripheral, or coronary arteries. Comparison with previous examinations is essential because chronic atherosclerotic aneurysms, postoperative pseudoaneurysms, sinus of Valsalva aneurysms, and stable anastomotic abnormalities may mimic infection. New morphology, rapid progression, surrounding inflammation, and concordant microbiological evidence increase diagnostic confidence.

Cardiac CT should also assess the coronary arteries and their relationship to periannular infection. Coronary complications may result from septic embolization, obstruction of a coronary ostium by infected tissue, extrinsic compression by an abscess or pseudoaneurysm, thrombosis, fistulization, or involvement of a previous bypass graft. Even when the coronary lumen remains patent, proximity of infection to the left main or right coronary ostium may substantially alter surgical planning [11,24,29].

The myocardium and pericardium should be reviewed for direct extension of infection, focal collections, inflammatory thickening, or pericardial effusion. CT can demonstrate established anatomical abnormalities, whereas cardiac MRI is more sensitive for myocardial edema, inflammation, and tissue characterization.

### 7.5. Coronary and Surgical Planning

Many patients referred for valve surgery require evaluation of coronary artery disease. Coronary CTA may allow simultaneous assessment of valvular infection, periannular complications, coronary stenosis, coronary ostia, bypass grafts, and the relationship of infected tissue to the coronary circulation [10,11,12,24].

CTA is most useful in patients with a low or intermediate probability of obstructive coronary disease and adequate rhythm, renal function, and image quality. Invasive angiography may remain necessary when the pretest probability is high, CT is nondiagnostic, extensive calcification or stents limit interpretation, or percutaneous treatment may be required. Coronary evaluation should not delay urgent surgery when the result is unlikely to alter management.

Preoperative CT is particularly valuable before repeat sternotomy. The examination should define the relationship of the sternum to the right ventricle, ascending aorta, pulmonary artery, bypass grafts, conduits, pseudoaneurysms, and infected collections. It should also assess graft patency and course, aortic calcification, previous surgical reconstruction, and potential peripheral cannulation pathways [24].

For complex periannular disease, the report should provide a surgical roadmap rather than merely state that infection is present. Relevant information includes the circumferential extent of annular involvement, dimensions and location of abscesses or pseudoaneurysms, fistulous communications, involvement of the intervalvular fibrosa or ventricular septum, proximity to the coronary ostia, and extension into previous grafts or conduits. These findings may determine whether surgery requires isolated valve replacement, annular reconstruction, aortic-root replacement, intervalvular fibrosa reconstruction, fistula closure, coronary reimplantation, or bypass grafting.

### 7.6. Clinical Integration and Limitations

Extracardiac and preoperative findings are clinically valuable only when they influence diagnostic confidence, surgical timing, procedural strategy, source control, or antimicrobial management. An incidental asymptomatic infarct does not carry the same importance as an abscess, unstable infectious aneurysm, intracranial hemorrhage, coronary obstruction, or collection adjacent to a repeat sternotomy route.

The principal limitations of extended CT assessment are ionizing radiation, iodinated contrast exposure, incomplete sensitivity for early cerebral and spinal disease, difficulty distinguishing sterile infarction from early infection, and the possibility of incidental findings that generate unnecessary investigation. Rhythm disturbance, extensive coronary calcification, renal dysfunction, and unstable physiology may additionally limit preoperative coronary CTA.

Anatomical coverage should therefore be tailored to symptoms, microbiology, previous surgery, renal function, prior imaging, and the expected management consequence. The Endocarditis Team should determine when additional imaging is justified and how cardiac and extracardiac findings alter the overall treatment strategy. Key extracardiac and preoperative CT targets and their clinical relevance are summarized in Table 5.

## 8. Emerging Diagnostic Technologies

### 8.1. From Improved Image Quality to Quantitative Imaging

Emerging cardiac CT and computational technologies differ substantially in both maturity and direct relevance to infective endocarditis. To avoid conflating technical feasibility with clinical validation, the evidence discussed in this section is categorized into four levels: (1) clinically validated IE-specific applications; (2) preliminary IE-specific evidence without definitive clinical validation; (3) indirect evidence extrapolated from broader cardiovascular imaging; and (4) conceptual applications without direct IE-specific evaluation. These categories describe the maturity and directness of the evidence rather than the intrinsic technical capability of each technology.

None of the emerging technologies reviewed in this section currently meets the threshold for an established, clinically validated IE-specific diagnostic application. Spectral CT and deep learning or super-resolution reconstruction are supported principally by broader cardiovascular imaging studies [18,19,20,24], while photon-counting CT is supported by indirect cardiovascular and phantom evidence together with limited preliminary IE-specific observations [15,27,28]. Radiomics and conventional artificial intelligence remain investigational in IE [16,17], whereas foundation-model, natural-language-processing, and federated-learning applications remain predominantly conceptual for this disease.

Improved spatial resolution, reduced image noise, artifact suppression, or computational performance should therefore not be interpreted as improved IE diagnosis or clinical outcome without dedicated validation. The available evidence, its directness to IE, and the present clinical applicability of these technologies are summarized in Table 6.

### 8.2. Spectral and Photon-Counting CT

Dual-energy and spectral CT provide attenuation information at different X-ray energy levels and enable reconstruction of virtual monoenergetic images, iodine maps, virtual non-contrast datasets, and material-decomposition images [38]. These techniques are potentially valuable in IE because low-energy reconstructions can increase iodine conspicuity, while higher-energy images may reduce beam-hardening and metallic artifacts from prosthetic valves, annuloplasty rings, transcatheter frames, pacing leads, and surgical material [24,28,38].

Spectral information may also assist in distinguishing enhancing inflammatory tissue from fluid, thrombus, or fibrosis. However, the attenuation and iodine characteristics of periannular infection, pannus, thrombus, and postoperative change overlap substantially. Spectral findings should therefore complement morphological interpretation rather than serve as stand-alone evidence of infection.

Photon-counting detector CT directly measures individual X-ray photons and categorizes them according to energy. Compared with conventional energy-integrating detectors, this technology can provide smaller detector elements, reduced electronic noise, improved dose efficiency, higher spatial resolution, and intrinsic multienergy information [15,38].

Initial clinical cardiovascular studies have demonstrated improved spatial resolution and reduced blooming, particularly around coronary calcification and stents. Mergen et al. showed improved anatomical sharpness using ultra-high-resolution photon-counting coronary CTA, while phantom investigations demonstrated reduced partial-volume and blooming effects [27,40]. These properties may be particularly relevant when IE involves prosthetic valves or heavily calcified structures.

Prosthetic-valve imaging provides a more direct potential application. Boccalini et al. demonstrated reduced blooming and metallic artifacts and improved conspicuity of simulated lesions using photon-counting CT compared with dual-energy dual-layer CT [28]. Importantly, lesions of approximately 1.5–2 mm remained difficult to identify, illustrating that improved detector technology does not eliminate the challenge of very small abnormalities.

Direct clinical evidence in IE remains extremely limited. A case reported by Asif et al. used full-cycle photon-counting CT in CIED-related endocarditis and demonstrated additional tricuspid-valve lesions beyond the major lead-associated vegetation identified on TEE [42]. This report is clinically illustrative but does not establish diagnostic accuracy or routine indications.

K-edge and multi-material imaging represent more experimental extensions of photon-counting technology. Selective visualization of specific prosthetic materials and future targeted contrast agents may eventually allow improved separation of metal, iodine, calcium, thrombus, and inflammatory tissue [28,38]. At present, these applications remain predominantly preclinical and should not be presented as established IE tools.

### 8.3. Advanced Reconstruction and Motion Analysis

Deep learning reconstruction algorithms can reduce image noise while preserving or enhancing edge definition. Tatsugami et al. demonstrated lower noise, higher contrast-to-noise ratio, and improved coronary sharpness compared with conventional hybrid iterative reconstruction [18]. Xu et al. similarly found substantial improvements in image quality, although these improvements did not significantly alter downstream CT-derived fractional-flow-reserve performance [19].

This observation is particularly relevant to IE. Improved image appearance does not necessarily translate into improved lesion detection or diagnostic classification. A sharper reconstruction may facilitate visualization of small anatomical structures, but no dedicated study has demonstrated that DLR improves sensitivity for vegetation, leaflet perforation, abscess, or prosthetic infection.

Super-resolution reconstruction may further reduce apparent blooming and improve visualization of small structures. Orii et al. reported improved stent visualization and larger assessable in-stent lumens using super-resolution DLR [20]. These findings suggest potential advantages around prosthetic material, but the evidence remains indirect.

Automated cardiac-phase registration and motion analysis could eventually quantify vegetation excursion, prosthetic rocking, dynamic pseudoaneurysm filling, or changes in fistulous tracts across the cardiac cycle. Such methods may also assist automated selection of optimal valve planes and cardiac phases. However, no validated IE-specific system currently performs these tasks.

### 8.4. Radiomics and Quantitative Phenotyping

Radiomics converts imaging data into quantitative descriptors of lesion shape, attenuation, texture, spatial heterogeneity, and higher-order image characteristics. In cardiac CT, radiomics has been investigated primarily for coronary plaque, perivascular adipose tissue, myocardial abnormalities, and intracardiac masses rather than infective endocarditis [16,17].

Potential applications in IE include differentiation of vegetation from thrombus, active periannular infection from postoperative fibrosis, and pannus from infected prosthetic tissue. Quantitative imaging features might also contribute to assessment of treatment response, embolic risk, or progression of periannular disease.

The major limitation is reproducibility. Radiomic features are strongly influenced by scanner type, tube voltage, contrast timing, section thickness, reconstruction kernel, motion, preprocessing, and segmentation methodology. Multicenter harmonization and external validation are therefore essential before any quantitative signature can be considered clinically useful [16,17].

At present, there is no externally validated cardiac CT radiomics model specifically for IE. Radiomics should consequently be described as a research strategy rather than a diagnostic tool currently available for routine practice.

### 8.5. Artificial Intelligence and Multimodal Models

Artificial intelligence is increasingly used in cardiovascular imaging for segmentation, quantification, classification, workflow optimization, and outcome prediction [43,44,45,46]. In IE, the most realistic near-term applications are likely to involve workflow support rather than autonomous diagnosis.

Potential near-term applications are primarily workflow-oriented, including valve or prosthesis localization, lesion measurement, longitudinal comparison, structured extraction of findings, and registration of CT with echocardiography or PET.

IE nevertheless presents a difficult environment for machine learning. The disease is relatively uncommon, anatomically heterogeneous, and distributed across native valves, surgical prostheses, TAVI devices, CIED leads, congenital material, and complex postoperative anatomy. Surgical and pathological reference standards are available only in a subset of patients, and lesion appearance changes during antimicrobial therapy. These characteristics substantially limit the size and quality of labeled training datasets.

No AI system has currently undergone sufficient external validation to provide autonomous diagnosis of IE. Models trained on a single institution, scanner, or patient population should therefore be considered investigational until tested across different centers, vendors, prosthetic materials, and clinical settings.

### 8.6. Foundation Models, Natural-Language Processing and Collaborative AI

Foundation models, natural-language processing, and federated learning may eventually support structured report extraction, multimodal data integration, registry development, and multicenter model training. However, the supporting literature derives from general radiology, medical imaging, or broader cardiovascular applications rather than validated IE populations [47,48,49,50,51,52,53]. Their relevance to infective endocarditis is therefore currently conceptual, and these approaches should be regarded as potential research infrastructure rather than as established diagnostic technologies. Any future clinical application will require IE-specific validation, external testing, transparent governance, and expert verification of model outputs.

### 8.7. Translation to Clinical IE Imaging

The central translational requirement for emerging technologies is evidence that technical improvement changes IE diagnosis or management. Future studies should therefore evaluate lesion detection and characterization, reproducibility, incremental value over established multimodality imaging, effects on surgical planning or treatment selection, and relevant radiation, contrast, workflow, or patient-outcome endpoints.

Among the technologies reviewed, photon-counting CT has a plausible technical rationale for IE because spatial resolution and prosthesis-related artifacts are genuine limitations of conventional CT. However, current evidence remains dominated by non-IE cardiovascular studies, phantom investigations, and isolated clinical reports [15,27,28,38,39,40,41,42,55]. Radiomics and AI remain less mature for IE because dedicated training and external-validation datasets are lacking, while foundation-model and federated-learning applications are currently conceptual [16,17,43,44,45,46,47,48,49,50,51,52,53,56,57].

Hybrid PET/MRI represents a separate emerging multimodality approach that combines metabolic information with morphologic, functional, and tissue-characterization capabilities. In a recent systematic review and single-center experience, hybrid PET/MRI was diagnostically useful in 9 of 12 patients evaluated for suspected endocarditis. However, current evidence remains limited, and larger prospective studies and standardized protocols are required before routine use in IE [54].

Accordingly, none of these emerging technologies should currently be presented as an established IE-specific diagnostic tool. Their clinical role should be defined by dedicated prospective validation rather than by technical performance alone.

## 9. Evidence Gaps and Future Research Priorities

### 9.1. Limitations of the Current Evidence Base

Despite the increasing incorporation of cardiac CT into contemporary infective endocarditis imaging, the supporting evidence remains heterogeneous. Most diagnostic studies are retrospective or single-center investigations, frequently involve relatively small cohorts, and often include selected patients referred for surgery. Consequently, advanced aortic-valve disease, prosthetic-valve infection, and periannular complications are overrepresented, whereas uncomplicated native-valve disease, right-sided infection, transcatheter-valve endocarditis, and CIED-related disease remain less comprehensively studied [10,12,13,14,30,31].

Substantial methodological heterogeneity further limits comparison among studies. CT examinations differ with respect to scanner generation, ECG synchronization, number of reconstructed cardiac phases, section thickness, contrast administration, reconstruction algorithm, and use of spectral or high-resolution techniques. Definitions of vegetation, inflammatory infiltration, abscess, pseudoaneurysm, fistula, dehiscence, and paravalvular leakage are also inconsistent. In some studies, anatomically distinct abnormalities are combined into composite outcomes, making lesion-specific performance difficult to interpret.

Reference standards represent an additional limitation. Surgical inspection provides the strongest available anatomical comparator but is restricted to patients undergoing intervention and may itself be affected by previous embolization, antimicrobial treatment, incomplete operative documentation, or differences between imaging and surgical terminology. Histopathological confirmation is even less frequently available. In medically managed patients, diagnostic adjudication may depend on clinical criteria and follow-up, producing incorporation bias when imaging findings contribute to the final diagnosis.

Timing also requires greater attention. Vegetations, inflammatory infiltration, abscesses, and metabolic activity can change after initiation of antimicrobial therapy. Diagnostic studies should therefore record the interval between CT, echocardiography, nuclear imaging, antimicrobial treatment, and surgery rather than assuming that all examinations represent the same disease state.

These limitations explain why apparently precise sensitivity and specificity estimates should not be interpreted as universal characteristics of cardiac CT. Future evidence should prioritize standardized, lesion-specific and substrate-specific evaluation rather than further accumulation of small heterogeneous retrospective cohorts.

### 9.2. Standardization of Acquisition, Lesion Definitions, and Reporting

The most immediate research priority is methodological harmonization. Before increasingly sophisticated technologies are evaluated, conventional cardiac CT must be performed and interpreted using reproducible terminology and protocols.

Consensus definitions are particularly important for periannular abnormalities. Inflammatory infiltration or phlegmon should be distinguished from a mature abscess; an abscess should be differentiated from a contrast-filled pseudoaneurysm; a fistula should require demonstration of a true communication; and structural prosthetic dehiscence should be separated from the functional severity of paravalvular regurgitation. Definitions should ideally be developed through multidisciplinary consensus and correlated with surgical and pathological findings [10,12,13,14,29,30,31].

Acquisition protocols also require harmonization. Prospective multicenter comparisons should determine the optimal balance among temporal coverage, spatial resolution, radiation dose, contrast volume, and diagnostic yield. Important variables include single-phase versus multiphase acquisition, reconstruction interval, section thickness, cardiac-phase selection, artifact-reduction strategy, and protocols adapted for right-sided disease or prosthetic material.

Standardized reporting terminology and a minimum set of clinically relevant CT findings should also be evaluated prospectively. Future studies should determine whether lesion-oriented reporting improves completeness, interobserver agreement, recognition of critical findings, communication with surgeons and cardiologists, or operative planning. The objective should be a concise and reproducible reporting structure rather than a rigid template.

Harmonized acquisition, lesion terminology, and reporting would also provide the foundation required for meaningful multicenter registries, radiomics studies, and AI development. Without standardization at the imaging level, increasingly complex computational analysis risks amplifying variability rather than improving diagnosis.

### 9.3. Prospective Multicenter and Substrate-Specific Validation

Future diagnostic studies should be prospective, multicenter, and explicitly lesion-oriented. Comparisons between CT and echocardiography should report results separately for vegetation, leaflet destruction, abscess, pseudoaneurysm, fistula, dehiscence, and other clinically distinct abnormalities rather than relying primarily on patient-level diagnosis.

Native-valve disease requires particular attention because the current literature is dominated by more advanced and surgically treated disease. Modern multiphase and high-resolution CT should be evaluated according to vegetation size and valve position to determine whether contemporary technology meaningfully reduces the sensitivity gap relative to TEE for small lesions.

Prosthetic-valve endocarditis remains an important target because structural abnormalities caused by infection overlap with pannus, thrombus, fibrosis, chronic paravalvular leakage, and postoperative change. Studies combining CT with metabolic imaging and surgical or pathological reference standards should determine which anatomical and metabolic patterns most reliably distinguish active infection from noninfectious prosthetic abnormalities.

TAVI-associated endocarditis requires dedicated evidence rather than extrapolation from conventional surgical prostheses. The diagnostic significance of leaflet thickening, frame-associated abnormalities, periprosthetic uptake, paravalvular defects, and periannular inflammation should be established prospectively. Similar dedicated studies are required for CIED infection, where isolated pocket infection, systemic CIED infection, lead-associated infective endocarditis, and concomitant valvular involvement should be analyzed as distinct but potentially overlapping clinical entities.

Right-sided and congenital substrates are also underrepresented. Future studies should include patients with tricuspid or pulmonary infection, complex congenital anatomy, conduits, and multiple implanted materials so that the role of cardiac CT is defined across the full clinical spectrum rather than predominantly in left-sided surgical disease.

### 9.4. From Diagnostic Accuracy to Clinical Impact

The next generation of studies should move beyond sensitivity and specificity. A technically accurate examination has limited clinical value unless it changes diagnostic confidence, treatment planning, or patient outcomes.

Relevant outcomes include whether cardiac CT changes classification from possible to definite or rejected IE, prevents unnecessary repeat imaging, identifies previously unrecognized periannular disease, modifies the urgency or extent of surgery, alters coronary or cannulation planning, identifies an extracardiac intervention target, or changes the decision regarding device extraction.

Outcome-oriented investigations should also assess radiation exposure, iodinated contrast burden, examination time, cost, accessibility, and potential delays in treatment. A comprehensive cardiac CT protocol that combines valvular, coronary, and preoperative assessment may reduce the need for multiple examinations, but this potential efficiency has not been adequately tested against alternative diagnostic pathways.

Health-economic analyses are particularly lacking. The relevant question is not simply whether cardiac CT adds another diagnostic test, but whether its use prevents additional procedures, reduces diagnostic uncertainty, facilitates earlier surgery, shortens hospitalization, or improves resource allocation. Cost-effectiveness should therefore be evaluated alongside diagnostic and clinical outcomes.

Longitudinal studies are additionally needed to determine whether CT has a role in monitoring selected structural complications during treatment. Routine serial CT would expose patients to unnecessary radiation and contrast, but follow-up imaging may be valuable in carefully selected patients with conservatively managed pseudoaneurysms, evolving periannular abnormalities, or complex postoperative disease.

### 9.5. Validation of Emerging Technologies and International Collaboration

Photon-counting detector CT, spectral imaging, advanced reconstruction, radiomics, and multimodal AI should be evaluated through a staged translational pathway rather than adopted on the basis of improved image quality alone. Current photon-counting evidence demonstrates technical advantages in cardiovascular imaging, but clinical IE-specific validation remains extremely limited [15,27,28,38,39,40,41,42,55].

For photon-counting computed tomography and spectral imaging, task-based studies should determine whether reduced blooming, improved spatial resolution, and material decomposition actually increase detection of small vegetations, prosthetic dehiscence, narrow fistulous tracts, or early periannular disease. Studies should compare these technologies with contemporary conventional CT rather than with obsolete scanner generations.

Radiomics requires even greater methodological discipline. Any proposed IE imaging biomarker should undergo acquisition harmonization, segmentation robustness testing, internal validation, independent external validation, and assessment of incremental value over conventional imaging and clinical variables. Models derived from small single-center datasets should not be interpreted as evidence of clinical utility [16,17].

AI research requires multicenter datasets that represent the diversity of valve types, prostheses, scanners, reconstruction methods, microbiology, and disease severity encountered in practice. Evaluation should include not only algorithmic performance but also radiologist performance with and without AI assistance, false-positive burden, calibration, failure on unfamiliar prostheses or artifacts, and the ability of clinicians to recognize incorrect model outputs [46,53].

Federated learning may facilitate collaboration among institutions without centralizing raw imaging data and is therefore particularly attractive for a relatively uncommon disease such as IE [50,51,52,56,57]. Nevertheless, distributed training does not solve differences in data quality, lesion definitions, reference standards, or institutional practice. Governance, cybersecurity, bias monitoring, external validation, and post-deployment performance assessment remain necessary.

An international imaging registry could provide the scale required to study uncommon substrates and advanced technologies. A standardized dataset should integrate clinical characteristics, microbiology, valve or device substrate, acquisition parameters, lesion-level imaging findings, multimodality results, treatment, surgery, pathology where available, and longitudinal outcomes. Such infrastructure would support not only diagnostic validation but also radiomics, AI, comparative effectiveness, and health-economic research.

## 10. Conclusions

Cardiac CT is a complementary component of contemporary multimodality assessment of infective endocarditis, with greatest value when detailed anatomical definition of periannular or periprosthetic disease, complex postoperative anatomy, coronary relationships, or preoperative mapping is required. Its contribution is lesion- and substrate-specific and should be interpreted together with echocardiography, microbiology, metabolic imaging when appropriate, and multidisciplinary clinical assessment [5,10,11,12,13,14,32].

ECG-synchronized multiphase acquisition and valve-oriented reconstruction improve evaluation of dynamic and structural abnormalities, but protocols should remain tailored to the clinical question so that diagnostic information is balanced against radiation exposure and iodinated contrast burden [10,12,13,24,25].

Emerging technologies should be interpreted according to evidence maturity. Spectral and photon-counting CT and advanced reconstruction may improve technical image quality, whereas radiomics and artificial intelligence may enable quantitative analysis; however, most supporting evidence remains indirect or preliminary, and foundation-model or federated-learning applications are currently conceptual for IE. Dedicated multicenter validation using IE-specific clinical endpoints is required before these approaches can be considered routine diagnostic tools.

Future work should prioritize standardized acquisition and lesion definitions, prospective substrate-specific studies, and demonstration that imaging changes management or patient outcomes. Within this framework, cardiac CT is most useful not as a replacement for echocardiography, but as a targeted anatomical tool integrated into the Endocarditis Team pathway.

## Figures and Tables

**Table 1:** Lesion- and Question-Specific Cardiac CT Acquisition and Reconstruction Strategy in Suspected Infective Endocarditis.

Diagnostic Target	Preferred Acquisition/Reconstruction Approach	Main Purpose and Limitation	Supporting References
Vegetation and leaflet motion	ECG-synchronized multiphase acquisition with thin-section reconstruction and cine assessment at approximately 5–10% R–R intervals	Improves assessment of mobility and leaflet interaction; very small or highly mobile vegetations may remain better depicted by TEE	[10,12,13,24]
Periannular abscess or inflammatory extension	Contrast-enhanced ECG-synchronized thin-section acquisition with valve-aligned multiplanar reformations	Defines the extent of periannular tissue involvement and relationship to adjacent structures; a full cardiac cycle may not always be required for predominantly static lesions	[10,12,13,14,24]
Pseudoaneurysm or fistulous communication	Multiphase ECG-synchronized acquisition with cine reconstruction	Demonstrates communication with cardiac chambers and dynamic contrast filling; increased temporal coverage may increase radiation exposure	[10,12,13,24]
Prosthetic dehiscence or rocking	Multiphase ECG-synchronized acquisition with prosthesis-aligned multiplanar and cine reconstructions	Demonstrates abnormal prosthetic motion and periannular separation; metallic artifacts may impair evaluation	[10,12,13,24]
Prosthetic-valve or device-related infection	Thin-section contrast-enhanced imaging with optimized reconstruction kernels; spectral or metal-artifact-reduction techniques when available	Improves visualization adjacent to prosthetic material and leads; no reconstruction technique completely eliminates small-lesion limitations	[18,19,20,24,27,28]
Right-sided IE	Contrast timing adapted to right-heart enhancement, including diluted contrast–saline or multiphasic injection strategies when appropriate	Reduces dense venous contrast artifacts and heterogeneous right-heart opacification	[13,24]
Coronary and preoperative assessment	ECG-synchronized coronary CTA integrated into the examination when clinically required	Provides coronary and surgical anatomical mapping; acquisition should be adapted to rhythm, heart rate, and the need for multiphase valve assessment	[12,13,24]
Extracardiac or embolic complications	Selective delayed thoracic or extended anatomical coverage according to symptoms and suspected complications	Allows detection of pulmonary or systemic infectious complications; increases radiation and contrast exposure	[5,12,24]

**Table 2:** Lesion-Specific Cardiac CT Manifestations and Complementary Imaging Role.

Structural Lesion	Principal Cardiac CT Appearance	Main Interpretive Limitation	Complementary Imaging Role	Supporting References
Vegetation	Irregular hypoattenuating or intermediate-attenuation mass or focal infected leaflet thickening	Small and highly mobile lesions may be missed; thrombus and degenerative lesions may mimic infection	TEE is generally more sensitive for mobility and small lesions	[10,12,13,31]
Leaflet perforation or destruction	Focal discontinuity, irregular leaflet contour, flail segment, or extensive tissue loss	Small defects and regurgitant flow may not be demonstrated	TEE and color Doppler confirm flow and hemodynamic significance	[10,13,31]
Valvular aneurysm	Dynamic saccular deformation or loss of normal leaflet curvature	May resemble vegetation or motion artifact	TEE better demonstrates dynamic tissue deformation	[12,30]
Inflammatory infiltration or phlegmon	Ill-defined periannular thickening, enhancement, or loss of normal tissue planes	Overlap with postoperative fibrosis and chronic inflammation	PET/CT or follow-up imaging may help determine activity	[12,14,29]
Abscess	Low- or fluid-attenuation periannular lesion with irregular enhancing inflammatory tissue	May coexist with pseudoaneurysm or remain predominantly solid	CT provides detailed anatomical mapping; TEE adds dynamic evaluation	[10,12,13,14]
Pseudoaneurysm	Contrast-filled perivalvular cavity communicating with a chamber or the aortic root	Postoperative cavities and vascular structures may mimic disease	Multiphase CT defines the neck and extent; TEE may demonstrate communication	[10,12,13,14,29,30]
Fistula	Continuous contrast-filled tract between cardiovascular structures	Small tracts may be missed or overcalled	TEE and color Doppler demonstrate active flow; CT maps the anatomical course	[12,29,30]
Prosthetic dehiscence	Gap, paravalvular contrast channel, sewing-ring disruption, malalignment, or rocking motion	Pledgets, chronic leakage, and metallic artifacts may mimic infection	CT maps circumferential extent; TEE quantifies regurgitation	[10,13,29,30]

**Table 3:** Principal Comparative Evidence for Cardiac CT and Transesophageal Echocardiography in Infective Endocarditis.

Evidence Source	Population/Reference Standard	Endpoint	Cardiac CT	TEE/Echocardiography	Main Interpretation
Oliveira et al. [10]	Meta-analysis; 8 studies; surgical findings	Abscess/pseudoaneurysm	Sens. 78% (95% CI 70–85%); spec. 92% (87–95%)	Sens. 69% (62–76%); spec. 96% (94–98%)	CT showed higher sensitivity; multiphase CT sensitivity reached 87% (78–93%)
Oliveira et al. [10]	Meta-analysis; surgical findings	Vegetation	Sens. 64% (57–70%); spec. 88% (80–94%)	Sens. 94% (92–96%); spec. 82% (74–89%)	TEE substantially more sensitive
Oliveira et al. [10]	Meta-analysis; surgical findings	Leaflet perforation	Sens. 41% (25–59%); spec. 92% (86–96%)	Sens. 81% (71–88%); specificity not consistently estimable	TEE more sensitive
Jain et al. [31]	Meta-analysis; 10 studies, 872 patients; surgical pathology	Periannular complications	Sens. 88.0% (72.1–95.4%); spec. 93.2% (87.3–96.5%)	Sens. 70.3% (58.8–79.7%); spec. 95.5% (90.1–97.8%)	CT showed a trend toward higher sensitivity
Jain et al. [31]	Meta-analysis; 10 studies, 872 patients; surgical pathology	Leaflet perforation	Sens. 48.4% (31.7–65.4%); spec. 92.7% (78.1–97.9%)	Sens. 79.1% (70.5–85.7%); spec. 92.7% (83.7–97.0%)	TEE significantly more sensitive
Koo et al. [13]	49 preoperative patients; surgical inspection	Multiple lesion types	Sens.: vegetation 90.9%; perforation 50.0%; abscess/pseudoaneurysm 60.0%; paravalvular leakage 50.0%	Sens.: 100%, 87.5%, 40.0%, and 100%, respectively	Lesion-specific complementary performance
Hryniewiecki et al. [14]	53 surgical patients, 71 affected valves	Abscess/pseudoaneurysm	Sens. 81%; spec. 90%	TTE + TEE: sens. 63%; spec. 90%	Combination detected all 11 surgically identified lesions in this cohort

**Table 4:** Substrate-Specific Role of Cardiac CT in Infective Endocarditis.

Clinical Substrate	Principal Role of Cardiac CT	Main Limitation	Complementary Modalities	Supporting References
Native aortic-valve IE	Assessment of the annulus, aortic root, intervalvular fibrosa, periannular extension, and coronary relationships	May miss small or highly mobile vegetations	TTE and TEE	[10,11,12,13,24,29,30]
Native mitral-valve IE	Characterization of leaflet destruction, annular involvement, and extension toward the intervalvular fibrosa or circumflex artery	Inferior to TEE for small perforations and regurgitant flow	TTE and TEE	[10,12,13,29,30]
Surgical prosthetic-valve IE	Sewing-ring assessment, abscess, pseudoaneurysm, fistula, dehiscence, and surgical mapping	Artifacts and overlap with pannus, thrombus, pledgets, and chronic postoperative changes	TEE, FDG PET/CT, and WBC-SPECT/CT	[1,11,12,23,24,28,29,32]
Transcatheter Aortic Valve Replacement (TAVR) -associated IE	Evaluation of the frame, leaflets, annular interface, and aortic-root complications	Leaflet thickening is nonspecific and may reflect thrombosis	TEE and FDG PET/CT	[5,24,29,33]
CIED infection/CIED-related IE	Lead-course mapping, larger intracardiac masses, venous complications, lead perforation, pulmonary septic complications, and extraction/procedural planning	Limited sensitivity for small lead-associated lesions; CT cannot reliably distinguish infected from sterile lead material	TTE, TEE, ^18^F-FDG PET/CT, and microbiology	[23,24,28,32]
Right-sided IE	Assessment of right-heart anatomy, device–valve relationships, and pulmonary septic complications	Contrast-mixing and device-related artifacts	TTE, TEE, and thoracic CT	[24,29,32]

**Table 5:** Extracardiac and Preoperative CT Assessment in Infective Endocarditis.

Target	Principal CT Contribution	Clinical Relevance	Main Limitation	Supporting References
Brain and intracranial vessels	Detection of hemorrhage, large infarction, abscess, or infectious aneurysm	May alter surgical timing and require neurological or endovascular intervention	MRI is more sensitive for small or early lesions; distal aneurysms may require catheter angiography	[5,11,24,29]
Spleen, kidneys, and abdomen	Identification of infarction, abscess, rupture, mesenteric ischemia, or bowel injury	May provide embolic evidence or require drainage, surgery, or altered treatment sequencing	Early infected infarction may resemble sterile infarction	[5,24,29]
Lungs and pulmonary arteries	Detection of septic nodules, cavitation, infarction, emboli, abscess, and pleural complications	Supports right-sided or device-related infection and may affect respiratory or surgical management	Findings are not specific for IE	[24,29,32]
Osteoarticular system	Detection of advanced spondylodiscitis, osseous destruction, or paravertebral collection	Identifies metastatic infection or a possible bacteremic source	MRI is more sensitive for early and epidural disease	[5,24]
Systemic and coronary arteries	Identification of infectious aneurysm, thrombosis, embolism, compression, or rupture	May require vascular intervention and influence valve-surgery timing	Chronic and postoperative aneurysms may mimic active infection	[11,24,29]
Coronary artery disease	Evaluation of coronary stenosis, ostia, stents, and bypass grafts	Determines the need for concomitant revascularization	Calcification, rhythm, and unstable physiology may reduce diagnostic quality	[11,12,24]
Redo-sternotomy anatomy	Mapping of the sternum, cardiac chambers, ascending aorta, grafts, conduits, and collections	Reduces re-entry risk and guides access and cannulation	Adhesions cannot be directly characterized with certainty	[11,12,24]
Periannular surgical roadmap	Definition of abscesses, pseudoaneurysms, fistulae, coronary relationships, and previous reconstruction	Determines the extent and type of surgical reconstruction	CT findings must be integrated with TEE and operative assessment	[10,12,13,14,24,29]

**Table 6:** Evidence Maturity and Current Clinical Applicability of Emerging Imaging and Computational Technologies in Infective Endocarditis. NLP, natural-language processing.

Technology	Evidence Category	IE Population Directly Studied?	Main Evaluated Endpoint	Principal Limitation	Supporting References
**Spectral/dual-energy CT**	Indirect cardiovascular evidence	No dedicated IE validation cohort	Virtual monoenergetic imaging, iodine characterization, artifact reduction	No validated IE-specific diagnostic endpoint; overlap among infection, thrombus, pannus, and postoperative change	[24,28,38]
**Photon-counting detector CT**	Preliminary IE-specific + indirect cardiovascular evidence	Only isolated IE reports; most evidence from cardiovascular cohorts and phantom studies	Spatial resolution, blooming reduction, prosthetic and calcified-structure visualization	No prospective IE diagnostic-accuracy validation; very small lesions remain difficult	[15,27,28,38,39,40,41,42]
**Deep learning/super-resolution reconstruction**	Indirect cardiovascular evidence	No dedicated IE validation cohort	Noise reduction, contrast-to-noise ratio, sharpness, visualization of small or metallic structures	Improved image quality has not been shown to improve IE lesion detection	[18,19,20]
**Radiomics**	Indirect cardiovascular evidence	No externally validated IE cohort	Quantitative tissue and lesion phenotyping	Small heterogeneous datasets, protocol dependence, lack of reproducible IE-specific features and external validation	[16,17]
**Conventional AI/automated analysis**	Indirect and conceptual evidence	No externally validated IE diagnostic model	Segmentation, quantification, workflow support, multimodal integration	Dataset scarcity, domain shift, inconsistent reference standards, absence of IE-specific outcome validation	[43,44,45,46]
**Foundation models, NLP, and federated learning**	Conceptual for IE	No	Report extraction, multimodal integration, multicenter model development	No direct IE validation; hallucination, bias, governance, privacy, and dataset heterogeneity	[47,48,49,50,51,52,53]
**Hybrid PET/MRI**	Preliminary IE-specific evidence	Yes, but in a small subgroup	Combined metabolic, morphologic, functional, and tissue-characterization information	Very small IE sample and lack of standardized prospective validation	[54]

## Data Availability

No new data were created or analyzed in this study. Data sharing is not applicable to this article.
